# A Conformationally Stable Acyclic β‐Hairpin Scaffold Tolerating the Incorporation of Poorly β‐Sheet‐Prone Amino Acids

**DOI:** 10.1002/cbic.202100604

**Published:** 2021-12-16

**Authors:** Vesna Stanojlovic, Anna Müller, Ali Moazzam, Arthur Hinterholzer, Katarzyna Ożga, Łukasz Berlicki, Mario Schubert, Chiara Cabrele

**Affiliations:** ^1^ Department of Biosciences University of Salzburg Hellbrunnerstrasse 34 5020 Salzburg Austria; ^2^ Department of Bioorganic Chemistry Faculty of Chemistry Wrocław University of Science and Technology Wybrzeże Wyspiańskiego 27 50-370 Wrocław Poland; ^3^ School of Chemistry College of Science University of Tehran P.O. Box 14155–6619 Tehran Iran

**Keywords:** cation−π interactions, CH−π interactions, circular dichroism, hairpins, NMR spectroscopy

## Abstract

The β‐hairpin is a structural element of native proteins, but it is also a useful artificial scaffold for finding lead compounds to convert into peptidomimetics or non‐peptide structures for drug discovery. Since linear peptides are synthetically more easily accessible than cyclic ones, but are structurally less well‐defined, we propose XWXWXpPXK(/R)X(R) as an acyclic but still rigid β‐hairpin scaffold that is robust enough to accommodate different types of side chains, regardless of the secondary‐structure propensity of the X residues. The high conformational stability of the scaffold results from tight contacts between cross‐strand cationic and aromatic side chains, combined with the strong tendency of the d‐Pro‐l‐Pro dipeptide to induce a type II′ β‐turn. To demonstrate the robustness of the scaffold, we elucidated the NMR structures and performed molecular dynamics (MD) simulations of a series of peptides displaying mainly non‐β‐branched, poorly β‐sheet‐prone residues at the X positions. Both the NMR and MD data confirm that our acyclic β‐hairpin scaffold is highly versatile as regards the amino‐acid composition of the β‐sheet face opposite to the cationic−aromatic one.

## Graphical abstract

**Open but tight**: Combining the d‐Pro‐l‐Pro type II′ β‐turn motif with cross‐strand Trp/Lys(Arg) interactions leads to a conformationally and thermally highly stable β‐hairpin that is not sensitive to the type of residues occupying the upper face. This β‐hairpin scaffold is suited to accommodate a variety of amino acids, including poorly β‐sheet‐prone ones, and it does not require backbone cyclization

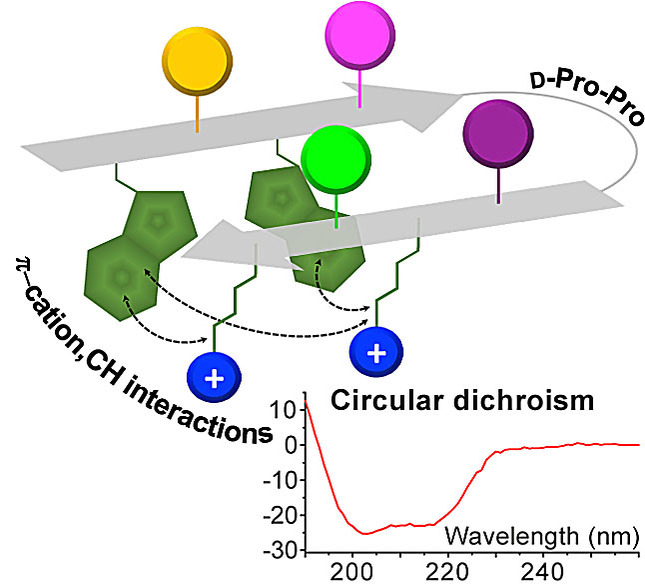

## Introduction

A β‐hairpin is a protein supersecondary structure that consists of two antiparallel β‐strands connected by a β‐turn or a short loop.^[1]^ A typical example of β‐hairpin is found in the C2H2 zinc‐finger, a DNA‐binding domain in which a β‐hairpin and a short helix are kept together by coordinating a zinc ion with two Cys thiols from the β‐hairpin and two His imidazole rings from the helix.^[2]^ Since β‐hairpins are involved not only in the folding of globular proteins[^1^, ^3^] but also in protein‐protein interactions,^[4]^ they have been intensively investigated.[^1^, ^5^] Moreover, a variety of chemical modifications have been used to achieve well‐defined β‐hairpin structures aiming at the mimicry of native β‐hairpins as well as at the development of peptide scaffolds and templates.^[6]^ About two decades ago, Robinson and co‐workers introduced a β‐hairpin scaffold consisting of a backbone‐cyclized peptide with d‐Pro‐l‐Pro as type II′ β‐turn‐inducer.^[7]^ This cyclic scaffold is particularly useful for the mimicry of short protein loops, and also for the development of synthetic antibiotics and vaccines.[^6j^, ^8^] In the late eighties, Mutter and co‐workers designed a backbone‐cyclized decapeptide containing two Pro‐Gly β‐turns that connect two three‐residue‐long arms.^[6g]^ This cyclic template has been used not only to assemble protein domains, like four‐helix bundles and β‐barrels,^[6g]^ but also to generate chemical diversity in combinatorial libraries.^[6e]^ Other strategies for the design of β‐hairpins take advantage of natural or chemically modified amino acids with β‐branched side chains that favor the formation of β‐strands.^[9]^ For example, Meldal, Schoffelen, and co‐workers^[10]^ developed small antibody mimetics (β‐bodies) consisting of a cyclopeptide containing the following structural elements: the β‐turn motif Pro‐Gly, the two arms with alternating Thr residues to favor an extended conformation,^[11]^ and side‐chain‐to‐side‐chain cyclization of the N‐terminal and C‐terminal residues by a triazole crosslinker.^[12]^

Besides cyclopeptides, linear peptides have also been proposed to mimic β‐hairpins: in this case, strong non‐covalent interactions between the two β‐strands in combination with a β‐turn motif have been exploited to compensate the lack of a macrocyclic structure. For example, Starovasnik and co‐workers developed the so called Trp zipper, in which two Trp−Trp cross‐strand pairs contribute to the stabilization of a short hairpin induced by a type I′ (Asn‐Gly) or II′ (Gly‐Asn or d‐Pro‐l‐Asn) β‐turn.^[13]^ Another example of cross‐strand π−π contacts is the β‐hairpin capping motif proposed by Andersen and co‐workers, which consists of the N‐terminal alkanoyl‐Trp residue and the C‐terminal triad Trp‐Thr‐Gly‐NH_2_: in this motif the two indole groups adopt a face‐to‐edge orientation, while the Thr side chain is involved in a H‐bond with the alkanoyl group.^[14]^ Further, Hughes and Waters used cross‐strand interactions between aromatic and positively charged groups:^[15]^ in detail, the contacts between a Lys residue on one arm and one or two Trp residues on the other arm were shown to stabilize β‐hairpins in combination with the type I′ β‐turn Asn‐Gly. Interestingly, Lys permethylation increased the thermal stability of the β‐hairpin because of the additional interactions of the methyl groups with the indole moiety, which strengthens the CH−π contact between the Lys ϵ‐CH_2_ group and the indole.[^15a^, ^16^] Also, two cross‐strand Trp−Lys pairs were found to form very stable β‐hairpins (so called WKWK) with high resistance against α‐chymotrypsin.^[17]^

**Figure 1:**
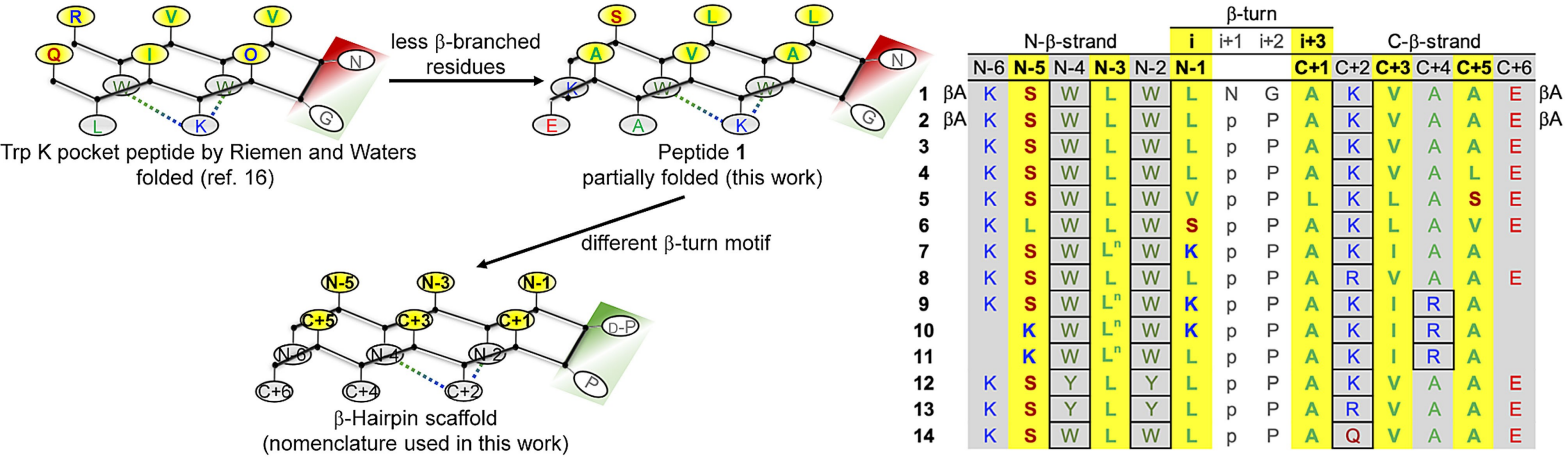
Overview of the synthetic peptides of this work obtained by modifications of the Trp K pocket β‐hairpin published by Riemen and Waters.^[16]^ The residues expected to be involved in π−π, cation/CH−π, and amide−π cross‐strand interactions are in the cells highlighted with black borders. All peptides are N‐terminally acetylated and C‐terminally amidated (βA=β‐alanine; L^n^=norleucine; O=ornithine; p=d‐proline).

While looking for an acyclic β‐hairpin scaffold that would be stable enough regardless of the type of residues displayed on one face of the β‐sheet, we selected the so called Trp K pocket,^[16]^ a hairpin featuring the type I′ β‐turn Asn‐Gly motif and the cross‐strand interaction between two Trp residues on the N‐terminal arm and a Lys residue on the C‐terminal arm (Figure 1). In fact, Riemen and Waters^[16]^ have previously shown by CD and NMR spectroscopy that the Trp K pocket peptide RWVWVNGOKILQ (O=ornithine; the Trp K pocket is underlined) is fully folded. Here, we performed amino‐acid substitution beyond the Trp K pocket and the turn, giving precedence to non‐β‐branched residues, which are less β‐strand prone than β‐branched ones.^[9]^ We show that the hairpin stability of the Trp K pocket scaffold WXWXNGXKX is affected by the nature of the residues at X positions. Therefore, we propose an improved scaffold, WXWXpPXK(/R)X(R) (p=d‐Pro), which is nearly independent of the presence of β‐branched residues along the arms of the hairpin, thus allowing a plethora of side chains to be displayed on one face of the β‐sheet.

## Results and Discussion

### The β‐hairpin scaffold based on the Trp K pocket and the type I′ β‐turn motif Asn‐Gly is affected by the nature of the residues within the strands

**Figure 2:**
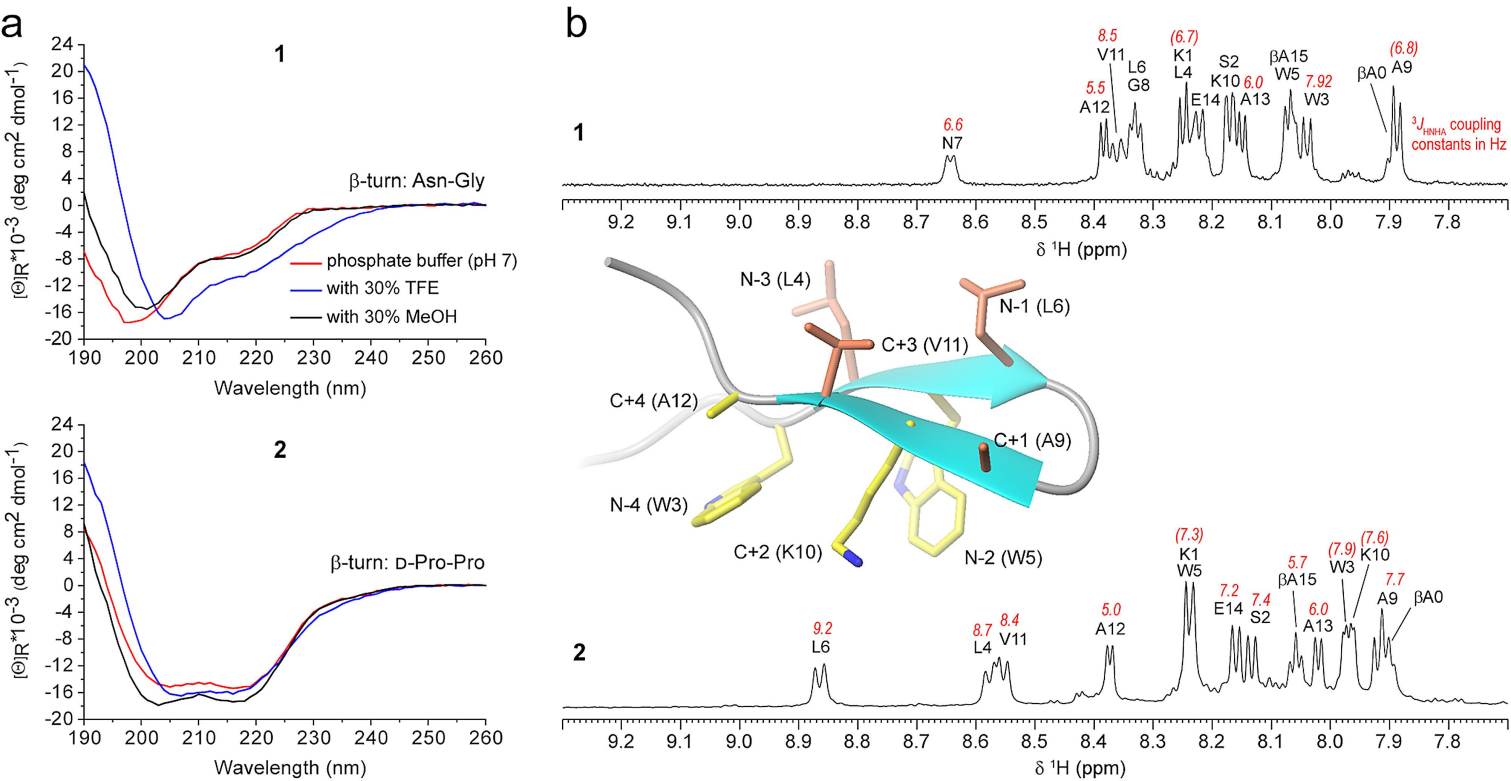
Impact of the β‐turn motif on the formation of a β‐hairpin based on the Trp K pocket. (a) CD spectra of peptide **1** at 58 μM (top), and gramicidin S‐like CD spectra of peptide **2** at 69 μM (bottom). (b) ^1^H NMR spectra of peptides **1** and **2** measured at 298 K in H_2_O/D_2_O with amide proton assignments and ^3^ *J*_HNHA_ scalar coupling constants. Values in brackets are estimates and could not be exactly extracted due to overlap. One of the ten low‐energy NMR solution structures of peptide **2** in water is shown as an example (the residue number is based on Figure 7, *vide infra*. The residues on the cationic−aromatic face are shown in yellow; the residues on the opposite face are shown in coral). The two Trp side chains are both found in the g+ rotamer (χ1≈−60°), so the aromatic rings are tilted towards the N‐terminus, thus exposing the two Hβ atoms towards the next residue.

To test whether the nature of the residues within the N‐terminal and C‐terminal strands would affect the conformational stability of the β‐hairpin scaffold based on the Trp K pocket and on the type I′ β‐turn motif Asn‐Gly, we modified the Trp K pocket peptide, a fully folded and thermally stable β‐hairpin published by Riemen and Waters,^[16]^ by reducing the number of the β‐strand‐prone β‐branched residues from three to one (Figure 1). The resulting peptide **1** is not well folded, despite its still high similarity with the Trp K pocket peptide mentioned above: in detail, the CD spectrum of the latter^[16]^ is characterized by an intense minimum close to 215 nm, indicative of β‐sheet structure, as well as by a moderate maximum close to 230 nm attributed to exciton interactions between the aromatic side chains.^[18]^ Instead, the CD spectrum of peptide **1** features a negative contribution at 215 nm, accompanied by a broad minimum below 200 nm, which is characteristic of unordered or irregular conformations^[19]^ (Figure 2a). The addition of secondary structure‐stabilizing cosolvents,^[20]^ like 2,2,2‐trifluoroethanol (TFE) and MeOH, did not help peptide **1** in adopting the fold of the Trp K pocket peptide: in fact, MeOH barely affected the shape of the CD curve, whereas TFE led to a minimum at 205 nm with a negative shoulder close to 215 nm and a maximum close to 190 nm, probably reflecting the formation of type I and III β‐turns.^[21]^

### Combination of the Trp K pocket with the type II′ β‐turn motif d‐Pro‐l‐Pro leads to a versatile β‐hairpin scaffold

We decided to replace the type I′ β‐turn motif Asn‐Gly in peptide **1** with the type II′ β‐turn motif d‐Pro‐l‐Pro, which was previously used in cyclic β‐hairpin scaffolds.^[7c]^ Moreover, d‐Pro at the i+1 position of a β‐turn has been shown to be better than Asn for the nucleation of a β‐hairpin.^[22]^ The CD signature of the new peptide **2** is, at first sight, quite surprising: indeed, the CD spectrum in phosphate buffer strongly resembles that of an α‐helix, but the bands fall at shorter wavelengths, with two minima of equally strong intensity at 218 nm and 204 nm, and positive CD contribution below 195 nm (Figure 2a, bottom, and Figure S1). This band shape persists after addition of cosolvents, although small shifts of the minimum over the range 203–207 nm have been detected. Since aromatic side‐chain contributions may hamper the interpretation of far‐UV electronic CD spectra,[^18d^, ^23^] we further used NMR spectroscopy to characterize peptide **2**. The ^1^H NMR spectrum of **2** shows significantly higher dispersion than that of **1**, with the number of downfield signals (>8.5 ppm) increasing from one to three, which is indicative of an increasing number of amides involved in H‐bonds (Figure 2b). Based on the chemical shifts of peptide **1**, the program TALOS+^[24]^ predicted a dynamic structure (data not shown), whereas many NOE correlations were found for peptide **2**, which enabled the calculation of its 3D structure, yielding a well‐folded β‐hairpin (Figure 2b). Then, we synthesized a variant of peptide **2** lacking the flanking β‐Ala residues that were initially thought to serve as linkers for future labeling of the hairpin. As expected, the new peptide **3** showed the same NMR structure and the same CD signature of peptide **2** (Figure S2). Thus, the CD spectrum of peptide **2** (Figure 2a, bottom) can be assigned to the folded β‐hairpin scaffold WXWXpPXKX. Noteworthy, this CD spectrum is very different from that of the Trp K pocket peptide based on the β‐hairpin scaffold WXWXNGXKX:^[16]^ in particular, we did not observe any positive CD contribution close to 230 nm, which is commonly attributed to exciton interactions between aromatic side chains,^[18d]^ as previously reported for the Trp K pocket,^[16]^ the Trp zipper^[18a]^ and other aromatic cages.[^18b^, ^18c^] Therefore, the two Trp side chains in our hairpin **2** are likely not as close as in the structures mentioned above: in fact, NOE contacts were hardly observed between the two indole moieties, whereas several NOE cross‐peaks were found between Lys10 (C+2) and each of the two Trp residues (Table S1), which indicates that the cationic side chain is positioned between the two aromatic groups. This is also reflected in the dramatic upfield chemical shift deviations of the Lys10 side chain induced by the ring currents of the two aromatic systems (*vide infra*). Additional backbone NOE contacts were observed between cross‐strand residues (i. e., Trp3/Ala12,13 and Trp5/Val11), which strengthens the presence of a well‐defined β‐hairpin (Figure S3). Interestingly, we found that the CD signature of our β‐hairpin **2** equals both in shape and intensity that of highly folded 14‐residue cyclic β‐hairpin analogs of gramicidin S containing an aromatic residue with d‐configuration at the i+1 position of one or both type II′ β‐turns (i. e., d‐Tyr‐l‐Pro).^[25]^ In general, gramicidin S and derived analogs display CD spectra with two minima over the range 200–220 nm and a maximum near 190 nm.[^25^, ^26^]

**Figure 3:**
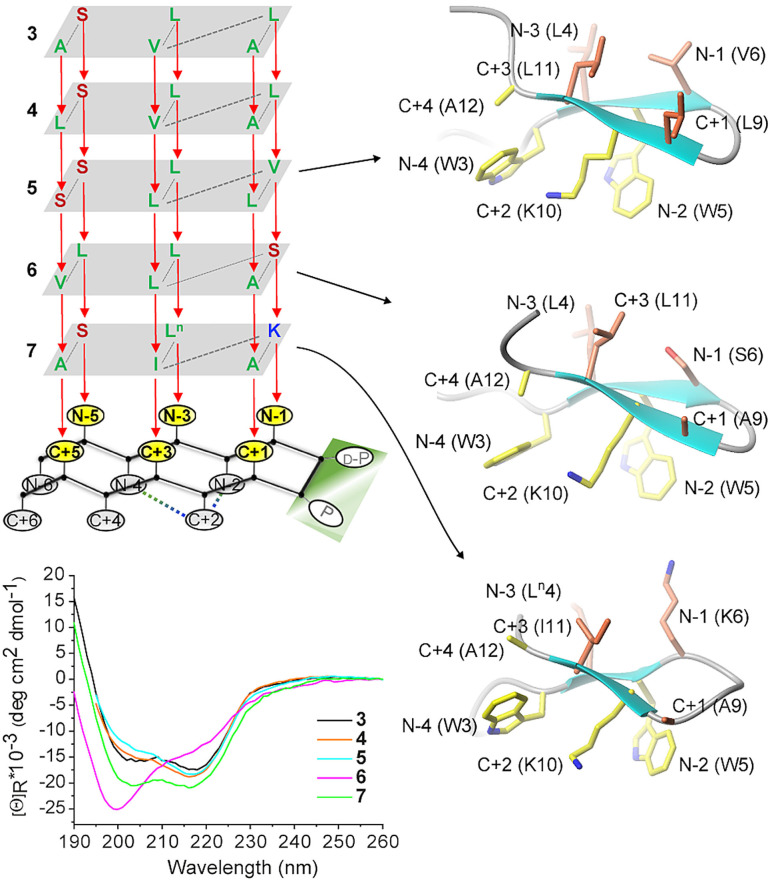
Versatility of the hairpin scaffold WXWXpPXKX for the assembly of different amino acids on the β‐sheet plate. Peptide concentrations were in the range of 36–61 μM. One of the ten low‐energy NMR solution structures of peptides **5**–**7** in water is shown as an example (the residue number is based on Figure 7, *vide infra*. The residues on the cationic−aromatic face are shown in yellow; the residues on the opposite face are shown in coral).

To test the versatility of the scaffold WXWXpPXKX for the assembly of different amino‐acid side chains on the hairpin face opposite to the cationic−aromatic face, we prepared four additional analogs of hairpin **3** (Figure 3). Except analog **6**, the other three analogs (**4**, **5** and **7**) display CD curves comparable to the dichroic signature of hairpins **2** and **3**, with the two minima near 218 nm and 204 nm and a positive contribution below 195 nm. In contrast, the CD spectrum of analog **6** shows reduced intensity of the minimum around 218 nm accompanied by a blue shift below 200 nm and enhanced intensity of the other minimum, suggesting partial destabilization of the hairpin structure. Accordingly, while peptides **5** and **7** show well‐dispersed ^1^H NMR signals, some of them even slightly more downfield shifted (>8.6 ppm) than those of peptide **2**, the ^1^H NMR spectrum of peptide **6** shows less dispersion and less downfield shifts (i. e., Leu4, Ser6, Leu11) than that of peptide **2** (Figure S4), suggesting the existence of an equilibrium between a folded β‐hairpin and an unfolded or partially folded population. Analog **6** is the only peptide containing a residue with a short polar side chain at position N‐1; since this position is part of the four‐residue β‐turn, it is plausible to think that the type of residue at N‐1 influences the β‐turn conformation. Moreover, as diagonal interstrand interactions have been reported to stabilize β‐hairpins,^[27]^ the diagonal interstrand positioning of Ser (N‐1) and Leu (C+3) side chains might be unfavorable.

### Conversion of the Trp K into a Trp R pocket confers both superior thermal stability and folding reversibility to the β‐hairpin scaffold

**Figure 4:**
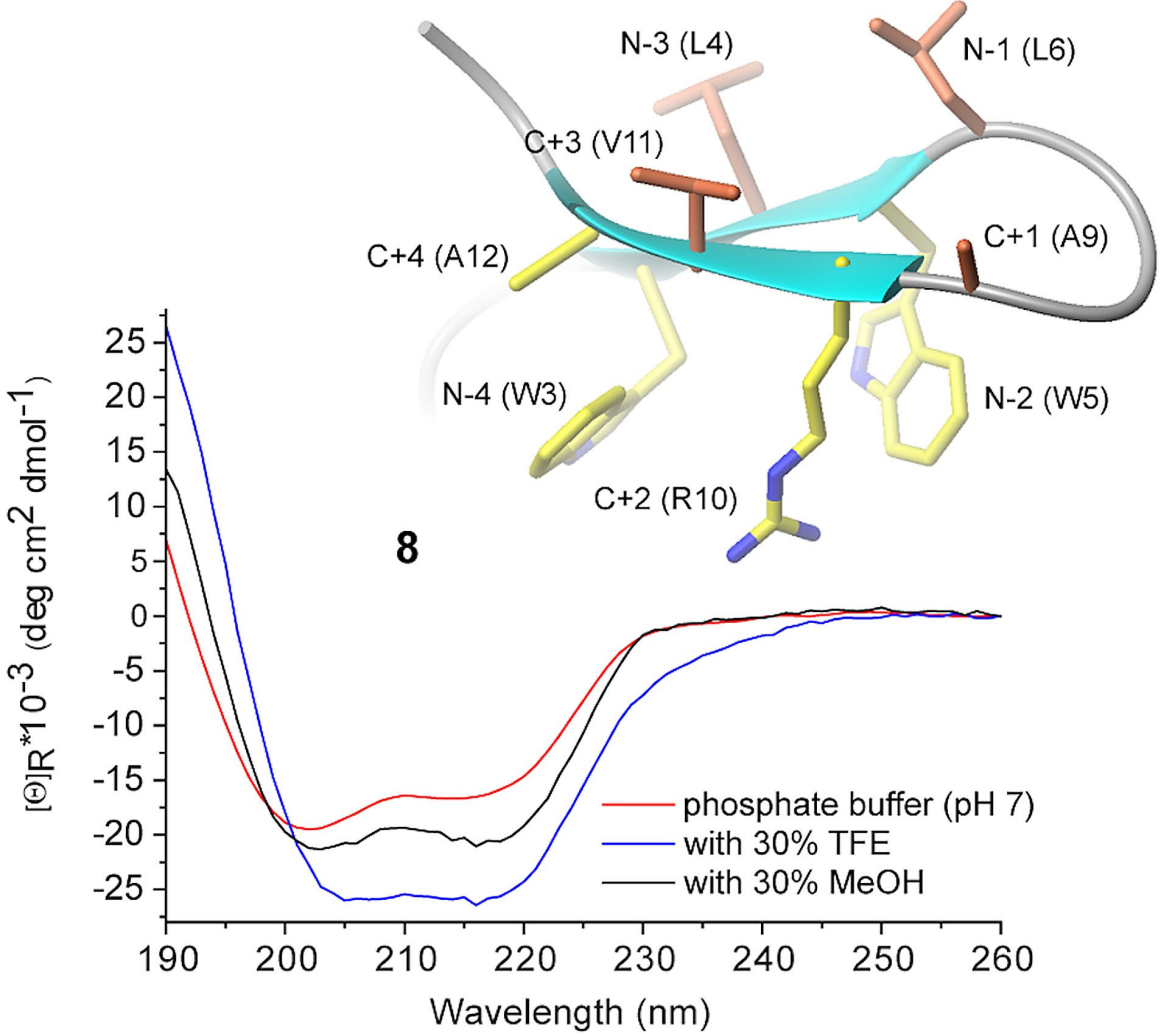
CD spectra of peptide **8** at 52 μM. One of the ten low‐energy NMR solution structures in water is shown as an example (the residue number is based on Figure 7, *vide infra*. The residues on the cationic−aromatic face are shown in yellow; the residues on the opposite face are shown in coral).

Since favorable cation−π and CH−π interactions with Trp side chains can occur not only for the Lys but also for the Arg side chain,[^27b^, ^28^] we replaced Lys at position C+2 with Arg: the resulting hairpin **8** features the same dichroic signature of the Lys‐containing analogs **2** and **3**, suggesting that the guanidinium group interacts as well with the Trp pocket (Figure 4). Interestingly, the cosolvents, particularly TFE, significantly increase the intensity of the CD bands, a phenomenon that has not been observed for the Lys‐containing analogs **2** and **3**, indicating that hairpin **8** undergoes further stabilization in a hydrophobic environment.

**Figure 5:**
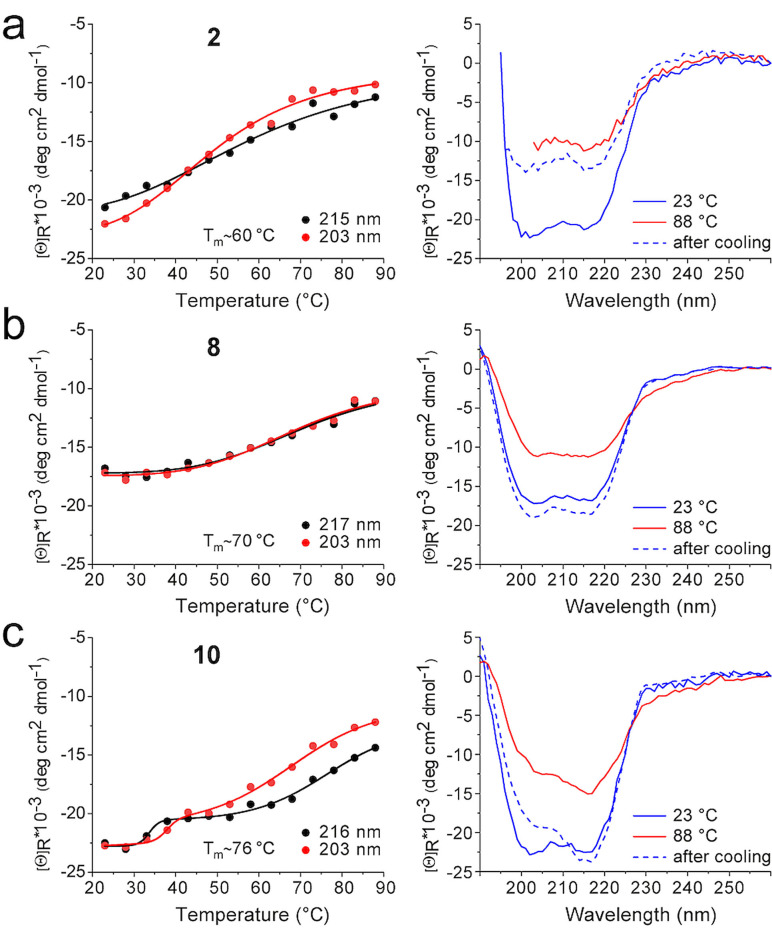
Comparison of the thermal stability of the peptide hairpins based on the Trp K/R (a/b) pocket, and aromatic/basic side‐chain zipper (c). In the panels on the left, the temperature dependence of the minima is shown. In the panels on the right, the CD curves before and after heating as well as after cooling are shown. Peptide concentrations were in the range of 31–68 μM.

We compared the thermal stability of the peptide hairpins based on the Trp K (**2**) and Trp R (**8**) pockets (Figure 5a,b). Both peptides underwent a conformational transition which, however, was reversible only for the Trp R pocket (**8**). This suggests a different type of interaction of the indole moiety with the Lys (**2**) and Arg (**8**) side chains: indeed, replacement of Lys with Arg not only increases the thermal stability (*T*_m_≈60 °C and 70 °C for **2** and **8**, respectively, based on the CD minimum at the longer wavelength), but also allows a reversible conformational transition of the hairpin. Another difference between the hairpins displaying cross‐strand Trp−Lys or Trp−Arg concerns the temperature dependence of the two CD minima: indeed, in the presence of Trp−Arg contacts (**8**), the two CD minima show the same temperature dependence, whereas in the presence of Trp−Lys contacts (**2**) the minimum at the shorter wavelength loses intensity faster than the minimum at the longer wavelength. Although the origin of the CD band at the shorter wavelength is unclear, the contribution of aromatic moieties is very likely;[^25^, ^26b^] thus, a tentative explanation might be that, in peptide **8**, Trp−Arg side‐chain rearrangement and backbone unfolding occur simultaneously, whereas, in peptide **2**, the Trp−Lys side‐chain rearrangement occurs before backbone unfolding. This would also fit with previous literature data, which report a moderately more favorable diagonal interaction energy of the Trp−Arg pair than that of the Trp−Lys pair.^[27b]^ Accordingly, comparison of the ^1^H NMR spectra of **2** (and **3**) and **8** (Figure S5) reveals that the amide signals for the residues involved in H‐bonds (Leu4, Leu6 and Val11) are slightly more downfield shifted in the case of **8**, suggesting that hairpin **8** is slightly more stable than hairpin **2**.

### Conversion the Trp K pocket into an aromatic/basic side‐chain zipper

**Figure 6:**
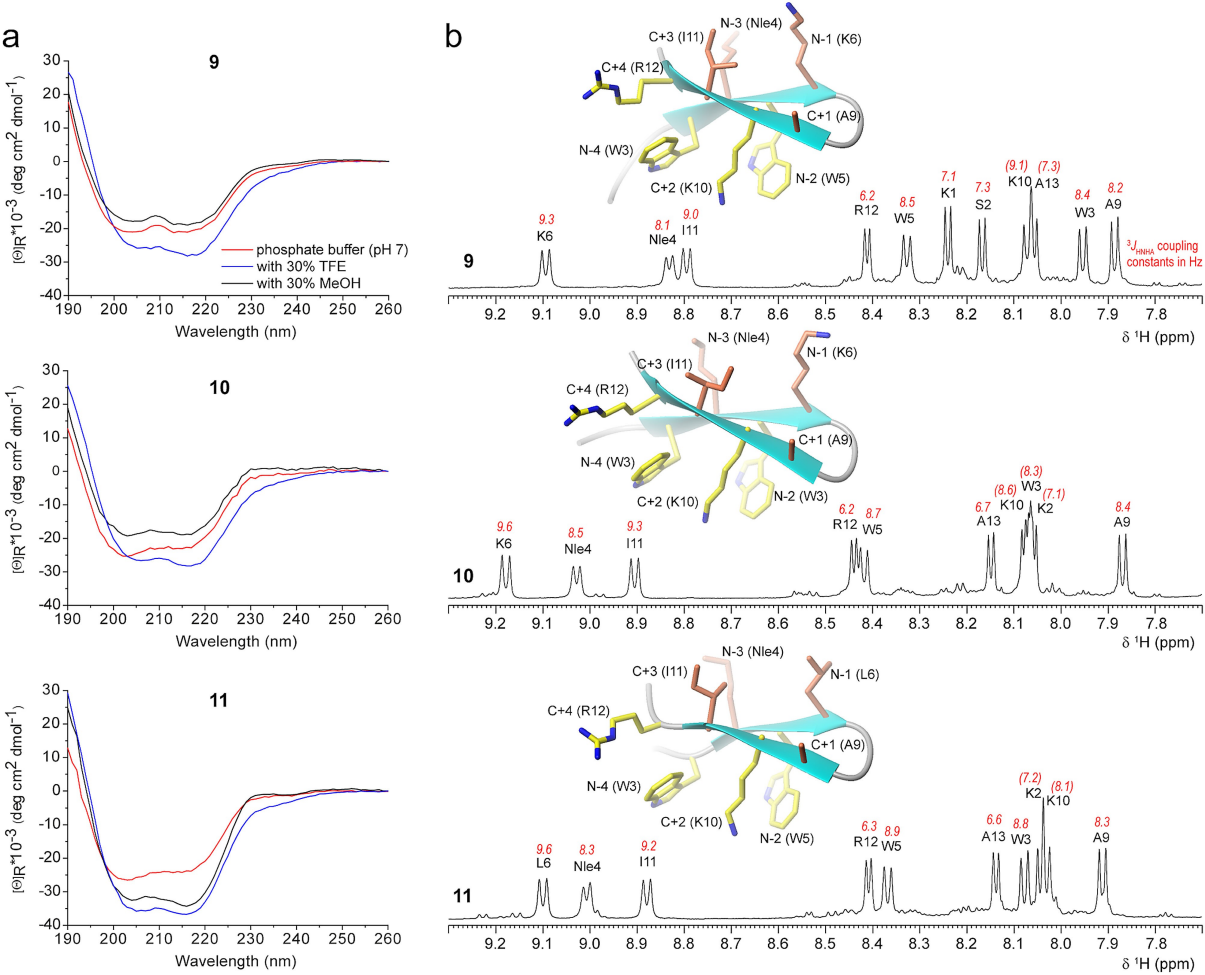
CD and NMR spectra of peptides **9**–**11** containing the aromatic/basic side‐chain zipper. (a) CD spectra measured with peptide concentrations of 67 μM (**9**), 39 μM (**10**), 70 μM (**11**). (b) ^1^H NMR spectra of peptides **9**–**11** measured at 298 K in H_2_O/D_2_O with amide proton assignments and ^3^ *J*_HNHA_ scalar coupling constants. Values in brackets are estimates and could not be exactly extracted due to overlap. One of the ten low‐energy NMR solution structures in water is shown as an example for each peptide (the residue number is based on Figure 7, *vide infra*. The residues on the cationic−aromatic face are shown in yellow; the residues on the opposite face are shown in coral).

Waters and co‐workers developed the so called WKWK β‐hairpin scaffold bearing two Trp residues in one arm and two Lys residues in the other arm, with the two arms connected by the β‐turn dipeptide Asn‐Gly or d‐Pro‐Gly.[^17^, ^29^] This WKWK hairpin is efficiently stabilized by two cross‐strand cationic−aromatic interactions. Therefore, we decided to introduce an additional basic residue at position C+4 in our scaffold, lateral to Lys at position C+2. Peptides **9**–**11** are all based on the expanded scaffold XWXWXpPXKXR that contains Arg at C+4. Their CD spectra (Figure 6a) are comparable to those of peptides **2**–**5**, **7**, and **8**, confirming that the introduction of an additional cross‐strand cationic−aromatic side‐chain interaction does not alter but rather stabilizes the β‐hairpin structure, as suggested by the slightly higher intensity of the CD spectra of **9**–**11** than those of **2**–**8**.

^1^H NMR spectra of peptides **9**–**11** (Figure 6b) show excellent dispersion and strongly downfield‐shifted amide signals for the residues involved in H‐bonds (residues 4, 6 and 11), which are comparable to fully folded cyclized β‐hairpins.[^25^, ^30^] The NMR solution structures of peptides **9**–**11** (Figure 6b) show that the two indole moieties and the Lys and Arg side chains are juxtaposed like the teeth of a zipper. Interestingly, the thermal unfolding of peptide **10** seems to consist of two distinct steps, the first one occurring already at 34–38 °C and the second one at 68–76 °C (Figure 5c). One tentative explanation might be that peptide **10** contains not only the Trp K pocket (Trp3−Lys10−Trp5), in analogy to peptides **2** and **8**, but also an additional cross‐strand cationic−aromatic side‐chain pair (Trp3−Arg12). Therefore, the early transition might reflect the loss of the side‐chain‐to‐side‐chain interaction Trp3−Arg12, whereas the late transition might reflect the loss of the Trp K pocket.

### Chemical shift deviations from random‐coil values reflect the conformational stability of the β‐hairpins

**Figure 7:**
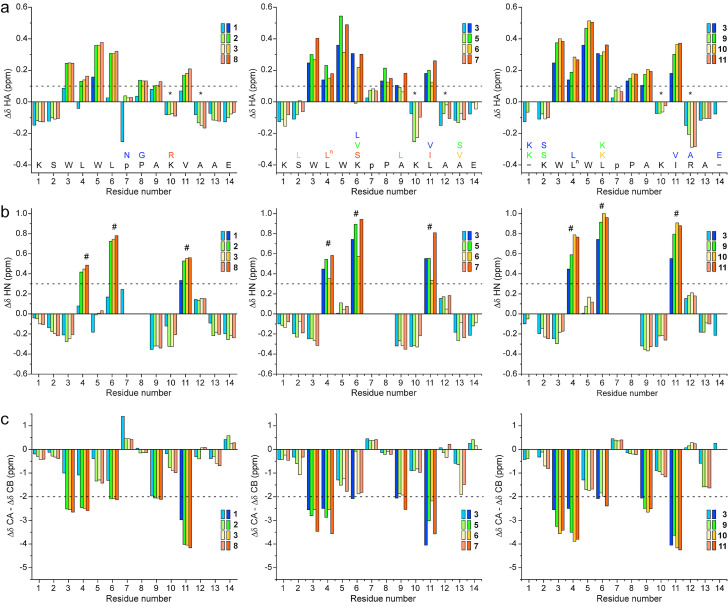
Backbone chemical shift deviations from random‐coil values.^[31]^ (a) Hα chemical shift deviations. Norleucine is indicated with L^n^ and d‐Pro with p. Protons displaying values larger than 0.1 (dashed line) are shown with a more intense color. The Hα protons of the residues indicated with an asterisk experience a strong upfield chemical shift due to ring currents of the Trp residues counteracting the downfield contribution of the peptide backbone in β‐strand conformation. (b) H^N^ chemical shift deviations. All amide chemical shifts with a deviation larger than 0.3 ppm (intense colors, above the dashed line) are involved in H‐bonds. (c) Chemical shift deviations of Cα and Cβ. Here [(Cα‐ Cα(r.c.))‐(Cβ‐Cβ(r.c.))] is plotted according to Marsh at al.^[32]^ (no smoothening was applied).

The complete NMR assignments of the peptides (Tables S2–13) allow the analysis of their secondary structure based on the deviations of the chemical shifts from random‐coil values (Δδ). Whereas peptide **1** shows no significant Hα chemical shift deviations (Figure 7a), peptide **2** displays important deviations for residues 3–6, 9 and 11. Positive values, particularly those >0.2 ppm, indicate β‐strand conformation. However, ^1^H chemical shifts are very sensitive to nearby aromatic rings. Ring‐current effects can obscure such an analysis of Hα chemical shift deviations, which is the case for the Hα deviations of residues 10 and 12 (indicated in Figure 7a by an asterisk). These Hα atoms are in the proximity of the indole ring and, thus, undergo a strong upfield shift, but they also lie in a β‐strand which causes a downfield shift. Therefore, the use of Hα deviations alone hampers the detection of β‐strands. In fact, we assume that the negative deviation of Lys10 (C+2) is the result of a strong positive deviation due to the β‐strand conformation and an even stronger negative effect from the ring current of Trp5 (N‐2).

Δδ(Hα) values are higher for peptides **5** and **7**–**11** than for peptides **2** and **3**, suggesting a better‐defined conformation. In fact, the Δδ(Hα) values between 0.3 and 0.6 ppm observed for peptides **5** and **7**–**11** are comparable to values reported for fully folded β‐hairpins stabilized by cyclization.[^27b^, ^34^] In contrast, peptide **6** shows slightly smaller deviations than peptide **2**, suggesting that its conformation is less defined, in agreement with the CD data.

An excellent indicator of involvement in a H‐bond is the H^N^ proton shift deviation from random‐coil values Δδ(H^N^) (Figure 7b), which quantifies the downfield shift seen in the ^1^H NMR spectra.

Whereas downfield shifts between 0.4 and 0.8 ppm are observed for Leu4, Leu6 and Val11 in peptide **2**, indicating the formation of H‐bonds (Figure S6), peptide **1** shows only one deviation barely higher than 0.3 ppm. Peptides **5** and **7**–**11** have higher Δδ(H^N^) values than peptides **2** and **3**, suggesting stronger H‐bonds. Some Δδ(H^N^) values are even in the range of 1 ppm, a value that is hardly reached in fully folded cyclic β‐hairpins,[^25^, ^27b^, ^34b^] suggesting the presence of 100 % folded form.

To avoid the interference of the strong ring‐current effects, we also analyzed the ^13^C chemical shifts of the Cα and Cβ atoms that are not significantly affected by the ring currents. In analogy to protein NMR, which typically predicts secondary structure from a plot of Δδ(Cα)‐Δδ(Cβ),^[35]^ strong negative values (<−2 ppm) are found for the β‐strands (Figure 7c).

**Figure 8:**
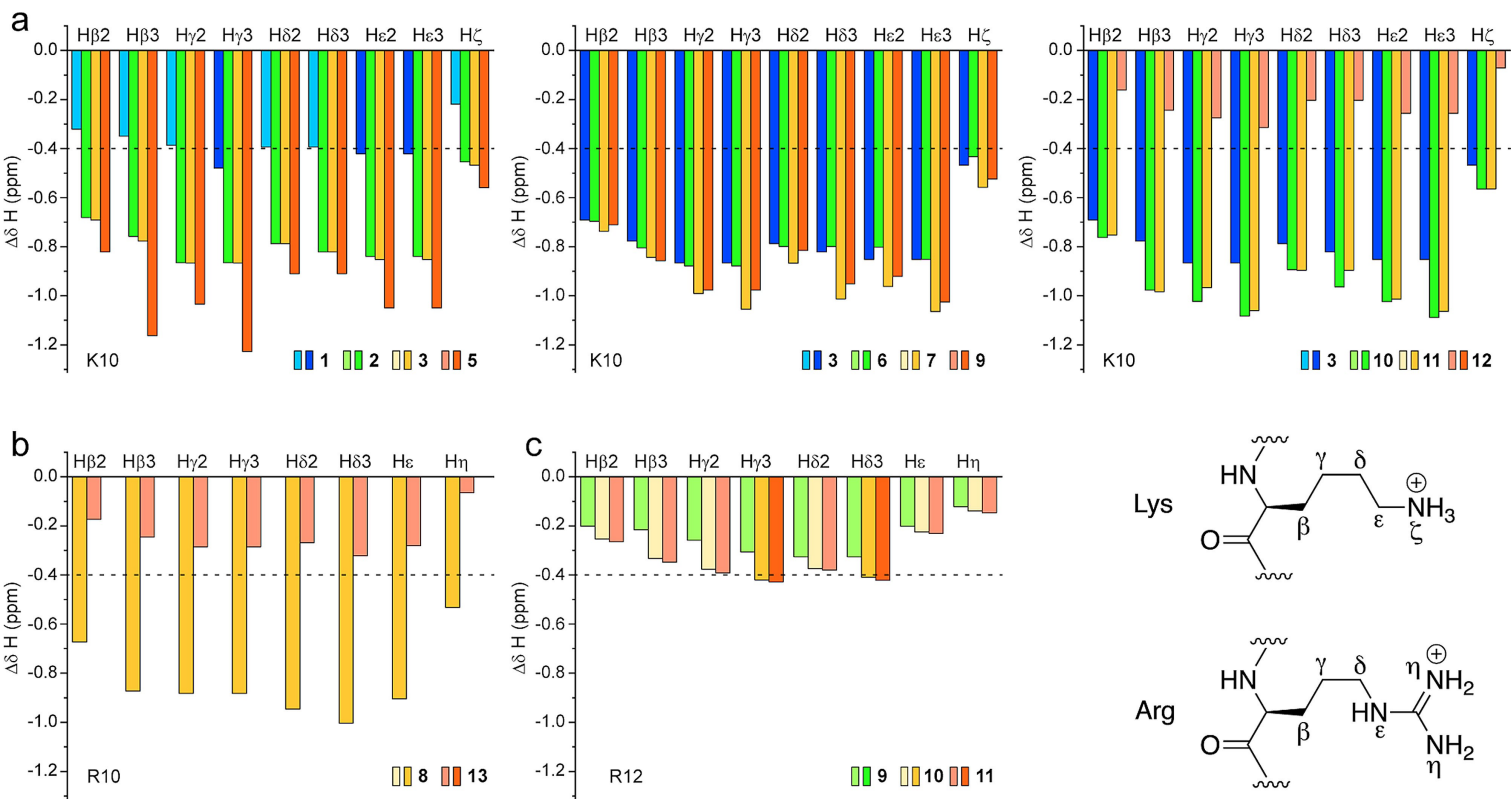
Chemical shift deviations from random‐coil values[^31^, ^33^] of the Lys (a) or Arg (b and c) side chain involved in cation/CH−π interactions. Protons displaying deviations larger than −0.4 ppm (dashed line) are shown with a more intense color.

The side‐chain chemical shift deviations of Lys10 from random‐coil values are more than doubled in peptide **2** compared to peptide **1** (Figure 8a), indicating a much stronger ring‐current effect of the two Trp residues (Trp3 and Trp5) and, thus, much closer Trp–Lys interactions in the d‐Pro‐l‐Pro‐containing peptide **2**. In peptide **8**, Arg10 displays even higher side‐chain Δδ(H) values than Lys10 in peptides **1** and **2** (Figure 8b), resulting from pronounced upfield shifts due to closer CH−π interactions.

The ^3^ *J*_HNHA_ scalar couplings extracted from the ^1^H NMR spectra are typical of β‐strand conformation (>8 Hz)^[36]^ for residues 3–6, 10 and 11 in peptides **6**–**8** (Figures S4 and S5). In contrast, residue 12 has ^3^ *J*_HNHA_ values below 8 Hz (5.0–5.4 Hz), which suggests a distortion of the β‐strand at this position.

For all peptides with the different amino‐acid variations on the β‐hairpin face opposite to the cationic−aromatic one, the upfield chemical shifts of the Lys10 side chain are comparable to those of Lys10 in peptide **3** (Figure 8a), indicating that the structure of the hairpin scaffold is conserved. Peptides **9**–**11** contain a second basic residue at position 12 (Arg12), which also shows upfield chemical shift changes of its side‐chain protons (Figure 8c), albeit with smaller magnitude. This indicates that Arg12 is in contact with Trp3 and experiences ring current from its aromatic moiety. However, the magnitudes are much smaller than for Lys10, suggesting that Arg12 is more dynamic than Lys10. ^3^ *J*_HNHA_ scalar couplings are >8 Hz for residues 3–6 and 9–11 in peptides **5** and **7**–**11** (Figures 6b, S4 and S5). Again, residue 12 shows ^3^ *J*_HNHA_ values below 8 Hz (around 5.4 Hz in peptides **5**–**7** and 6.2 Hz in peptides **9**–**11**), suggesting a distortion of the β‐strand at this position.

Replacement of both Trp residues by Tyr (peptides **12** and **13**) led to much less signal dispersion in the ^1^H NMR spectra, and signals above 8.5 ppm are absent (Figure S5). Although the Δδ(Hα), and Δδ(Cα)‐Δδ(Cβ) chemical shift deviations still show a trend towards β‐sheet conformation, the deviations are much smaller and mostly below the threshold, suggesting that the β‐sheet conformation is low‐populated in peptides **12** and **13** (Figure S7). The Δδ(H^N^) plot is even clearer, as it suggests no H‐bond formation for any of the residues (only for Leu6 of peptide **13** the value is barely above 0.3 ppm, as shown in Figure S7b). The upfield chemical shift deviations for the side chains of Lys10 (peptide **12**) and Arg10 (peptide **13**) are small and below the threshold (Figure 8a, b), indicating only transient interactions between residue 10 (Lys or Arg) and the two Tyr side chains.

### Molecular dynamics simulations confirm the high occurrence of cross‐strand cationic−aromatic interactions

To further prove the high conformational stability of the β‐hairpins, we performed molecular dynamics (MD) simulations. All the analyzed peptides (**2**, **5**–**11**) retain a compact and very stable conformation, as shown by the potential energy that was constant during the 5 ns long simulations (Figure S8). Moreover, close cross‐strand contacts between the ammonium/guanidinium ion of Lys/Arg and the benzene ring of the indole moiety of Trp are present for most of the simulation time (Figure S9 and Table S14), which further confirms the substantial contribution of this type of interaction to the conformational stability of the β‐hairpins studied.

## Conclusion

**Figure 9:**
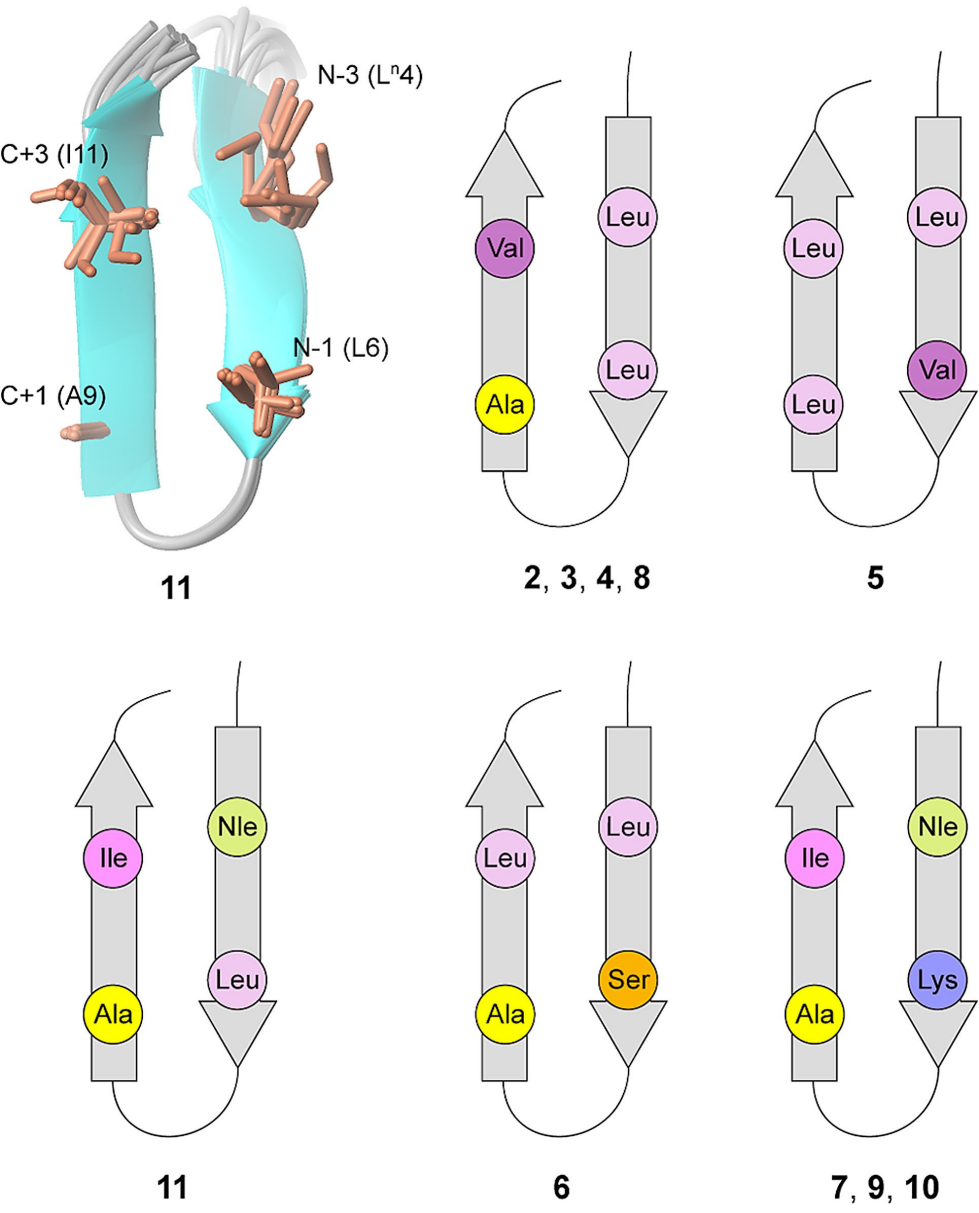
The versatile face of the β‐hairpin scaffold XWXWXpPXK(/R)X(R). The displayed side chains in the different peptides are represented by circles (for peptide **11** the ensemble of ten low‐energy NMR solution structures is also shown. The residue number is based on Figure 7, *vide supra*).

Starting from the concept of using the Trp K pocket to obtain well‐defined β‐hairpin structures,^[16]^ we developed an acyclic β‐hairpin scaffold that well tolerates the presence of different types of residues within the β‐strands. Most importantly, the content of β‐sheet prone residues, like the β‐branched ones, can be kept low, while residues usually preferring the α‐helix conformation, like Ala and Leu, are well tolerated and can be displayed on the β‐sheet face opposite to the Trp K(/R) pocket (Figure 9).

We found that the β‐turn motif plays a key role in uncoupling the conformational stability of the β‐hairpin from the secondary‐structure preference of the individual residues along the β‐strands: in fact, the type I′ β‐turn motif Asn‐Gly did not work properly and, thus, was replaced with the type II′ β‐turn motif d‐Pro‐l‐Pro, which resulted in the general scaffold XWXWXpPXKX. The latter was thermally stable with a *T*_m_ close to 60 °C, but the conformational transition was not reversible. However, after replacing Lys with Arg, we obtained a scaffold that is thermally highly stable (*T*_m_ near 70 °C) and can fold and unfold in a reversible manner.

Further, by introducing an additional basic residue at position C+4, lateral to Lys at C+2, we converted the Trp K pocket into an aromatic/basic side‐chain zipper with high thermal stability, which resulted indeed in a melting temperature near 76 °C for the peptide hairpin **10** based on the scaffold XWXWXpPXKXR.

Not only Trp−Lys/Arg but also Tyr−Lys/Arg pairs have been found to build energetically significant cation−π interactions in proteins.^[28]^ However, while replacing Trp with Tyr (peptides **12** and **13**), the hairpin scaffold was dramatically weakened, as reflected by the ^1^H NMR spectra with collapsed dispersion (Figure S5) and low‐intensity CD spectra (Figure S10). This agrees with data reported in the literature, which support the energetically stronger contribution of Trp residues rather than Tyr residues in cation−π interactions.^[18b]^

Besides cation−π interactions, also amide−π interactions are recurrent in molecular recognition events:^[37]^ a recent analysis of protein‐ligand interactions included in the Protein Data Bank^[38]^ revealed 2577 cation−π and 4967 amide−π contacts out of 750873 contacts.^[39]^ However, most of the amide−π interactions involve backbone rather than side‐chain amide groups, although there are examples of amide−π interactions involving acylated Lys residues.^[37a]^ We prepared peptide **14** containing Gln in place of Lys/Arg at position C+2. In comparison to the hairpins based on the Trp K(/R) pocket, this peptide (**14**) was characterized by more negative CD intensity below 200 nm, indicating the presence of an unfolded fraction that was reduced upon addition of cosolvents (Figure S11). Therefore, the amide−π stacking between the Trp side chains and the γ‐amide group of Gln seems to be less efficient than the cation/CH−π interaction in stabilizing the β‐hairpin. All together, these results show that a synthetically easily accessible, conformationally and thermally stable, versatile β‐hairpin scaffold can be achieved by the combination of interstrand Trp−Lys(/Arg) contacts with the type II′ β‐turn dipeptide d‐Pro‐l‐Pro.

## Experimental Section

**Peptide synthesis**: The peptides were assembled on an automatic peptide synthesizer (Syro I, Biotage) by using a Rink‐amide resin and Fmoc chemistry. The side‐chain protecting groups were tBu for Tyr, Boc for Lys and Trp, Pbf for Arg, Trt for Gln. The Fmoc deprotection was carried out with 25 % piperidine in DMF/NMP (70 : 30, v/v) for 3 min and 12.5 % piperidine in DMF/NMP (70 : 30, v/v) for 12 min. The couplings were accomplished with the mixture Fmoc‐AA‐OH/HOBt/HBTU/DIPEA (5 : 5 : 4.8 : 10 equiv.) for 2x40 min. The N‐terminal Lys residue of peptides **2**–**14** was coupled manually by performing a first coupling with the mixture Fmoc‐Lys(Boc)‐OH/HOBt/HBTU/DIPEA (10 : 10 : 9.8 : 20 equiv.) for 1 h, followed by a second coupling overnight with the mixture Fmoc‐Lys(Boc)‐OH/HOBt/DIC (10 : 10 : 10 equiv.). N‐terminal acetylation was performed manually with acetic anhydride/DIPEA (10 : 10 equiv.) in DMF for 30 min. The peptides were cleaved from the resin with TFA/H_2_O/TIA/EDT/TIS (90 : 1 : 3 : 3 : 3; V_tot_=1 ml) for about 3 h, precipitated by ice‐cold diethyl ether and recovered by centrifugation at 4 °C for 5 min. The homogeneity and identification of the lyophilized peptides were assessed by analytical HPLC (Thermo Fisher Scientific) and MALDI‐TOF‐MS (Bruker Daltonics) (Table S16 and Figures S12 and S13).

**CD spectroscopy**: The peptides were dissolved in 50 mM phosphate buffer (pH 7.3) with or without 30 % cosolvent (TFE or MeOH). The peptide concentrations were determined by the UV absorbance of Trp or Tyr using the molar extinction coefficients 5540 M^−1^ cm^−1^ or 1480 M^−1^ cm^−1^ at about 280 nm.^[40]^ The CD spectra were recorded on a Chirascan Plus spectrometer (Applied Photophysics) at 23 °C using a 1 mm quartz cell (Hellma Analytics). The *T*‐scans were recorded using a 0.5 mm quartz cell (Hellma Analytics). For each CD spectrum, three scans were accumulated using a step resolution of 1 nm, a bandwidth of 1 nm, and a time‐per‐point of 1 s. The CD spectrum of the solvent was subtracted and the difference spectrum was normalized to express the ellipticity in mean‐residue molar ellipticity, divided by 10^3^ and represented in the graphs as [θ]_R_×10^−3^ (deg cm^2^ dmol^−1^).

**NMR spectroscopy**: The NMR spectra were recorded on a 600 MHz AVANCE III HD spectrometer (Bruker Biospin) equipped with a QXI (^1^H/^13^C/^15^N/^31^P) probe at 298 K. Samples were dissolved in either D_2_O (100 atom%D, Armar Europe) or 7 % D_2_O/93 % H_2_O and measured in 5 mm NMR tubes (Armar Europe). Standard 2D NMR spectra were recorded: ^1^H‐^1^H TOCSY spectra with a mixing time of 120 ms, 1024×256 complex points, spectral widths of 12.9 ppm and 8.33 ppm, 4 scans and a recycle delay of 1 s; an identical experiment with a mixing time of 12 ms served as a ^1^H‐^1^H COSY; a ^1^H‐^1^H ROESY with a mixing time of 200 ms, 1024×350 complex points, spectral widths of 12.9 ppm and 10.0 ppm, 80 scans and a recycle delay of 1.2 s; a ^1^H‐^13^C HSQC optimized for aliphatic side chains with 512×125 complex points, spectral widths of 13.9 ppm and 77.0 ppm, 80 scans, a recycle delay of 1.5 s and a ^13^C offset of 45 ppm; a ^1^H‐^13^C HSQC optimized for aromatic side chains with 512×50 complex points, spectral widths of 13.9 ppm and 44.2 ppm, 80 scans, a recycle delay of 1.5 s and a ^13^C offset of 128 ppm. For some peptides, a ^1^H‐^15^N HSQC spectrum was measured using 512×64 complex points, spectral widths of 13.9 ppm and 36.5 ppm, 256 scans, a recycle delay of 1 s and a ^15^N offset of 116 ppm. Spectra were referenced to 2,2‐dimethyl‐silapentane sulfonic acid (DSS) using an external sample of 0.5 mM DSS and 2 mM sucrose in H_2_O/D_2_O. Data were processed with Topsin 3.6 (Bruker Biospin) and analyzed using Sparky 3.115 (T. D. Goddard and D. G. Kneller, SPARKY 3, University of California, San Francisco).

**NOE‐restrained structure calculations**: NOE cross‐peaks extracted from 2D ROESY spectra were categorized in small, medium, and strong, and converted into distance restraints. NOE upper limit restraints were applied based on signal‐to‐noise ratios of cross‐peaks extracted from 2D ROESY spectra using Sparky. Additionally, H‐bonds between residues N‐3 and C+3, C+3 and N‐3, N‐1 and C+1 were included as restraints at later stages in the calculations (Figures 7b and S6). Structure calculations were conducted with the Xplor‐NIH software^[41]^ using a standard protocol with the following steps: a) high temperature dynamics (3500 K, 800 ps or 8000 steps), b) simulated annealing performed from 3500 K to 25 K with 12.5 K step, at each temperature a short dynamics simulation was done (100 steps or 0.2 ps), c) gradient minimization of final structure. Finally, the ten top lowest energy structures were superimposed (Table S15). The structures were analyzed, fitted, and illustrated by using MolMol.^[42]^

**Molecular dynamics (MD) simulations**: 5 ns long MD was performed with the GROMACS software available on WCSS (Wrocław) using the Amber03 force field extended to non‐canonical amino acids and the lowest energy structure derived from NMR restraints as a starting point. Simulation box definition and solvation of the system were performed using the gmx editconf and the gmx solvate methods, and the spc216 water model. The minimization of the system was performed by using the steepest descent algorithm with 5000 maximum number of steps and PME electrostatics until F_max_<1000 kJ mol^−1^ nm^−1^. For all peptides the convergence was achieved in less than 700 steps. Equilibration of the system consisted of two phases: 100 ps (50000 steps) under the canonical ensemble (*NVT*) and 100 ps (50000 steps) under the *NPT* ensemble for temperature and pressure stabilization, followed by 5000 ps (2500000 steps) production run. All the dynamics were performed under periodic boundary conditions using the leapfrog scheme and PME electrostatics with a 1 nm cut‐off at a constant temperature of 300 K and a pressure equal to 1 bar. The trajectory analysis was performed using built‐in GROMACS protocols. To evaluate the statistics of cross‐strand cationic−aromatic side‐chain interactions, the trajectories of the distances between the Nζ/Nϵ of Lys/Arg at position C+2 or C+4 and the center of the benzene ring of the Trp indole moiety at position N‐2 or N‐4 were recorded (Figure S9); distances shorter than 6 Å were considered cross‐strand cation−π contacts and were expressed in time percentages (Table S14).

## Conflict of interest

The authors declare no conflict of interest.

1

## Supporting information

As a service to our authors and readers, this journal provides supporting information supplied by the authors. Such materials are peer reviewed and may be re‐organized for online delivery, but are not copy‐edited or typeset. Technical support issues arising from supporting information (other than missing files) should be addressed to the authors.

Supporting Information

## Data Availability

The data that support the findings of this study are available in the supplementary material of this article.
