# Supplementary material for: A Conformationally Stable Acyclic β‐Hairpin Scaffold Tolerating the Incorporation of Poorly β‐Sheet‐Prone Amino Acids
Source: Chembiochem. 2021 Dec 16;23(4):e202100604. doi: 10.1002/cbic.202100604 (PMC9299858; doi:10.1002/cbic.202100604)
Supplement: Supplementary file 1 — Supporting Information [file CBIC-23-0-s001.pdf]

ChemBioChem

Supporting Information

A Conformationally Stable Acyclic β-Hairpin Scaffold Tolerating the Incorporation of Poorly β-Sheet-Prone Amino Acids

Vesna Stanojlovic[+], Anna Müller[+], Ali Moazzam[+], Arthur Hinterholzer, Katarzyna Ożga, Łukasz Berlicki, Mario Schubert,* and Chiara Cabrele*

Materials

Chemical reagents and solvents for the peptide syntheses were of peptide-synthesis grade; solvents for HPLC and spectroscopy were of HPLC or spectroscopy grade. Fmoc-protected amino acids, Rink-amide MBHA resin (100-200 mesh, loading 0.57 mmol/g), N,N-diisopropylethylamine (DIPEA), piperidine, N,N-dimethylformamide (DMF), N-methyl-2-pyrrolidone (NMP), dichloromethane (DCM), diethylether and trifluoroacetic acid (TFA) were purchased from Iris Biotech (Germany). Thioanisole (TIA), acetic anhydride, acetonitrile, α-cyano-4-hydroxycinnamic acid, triisopropylsilane (TIS) and 1,2-ethanedithiol (EDT) were purchased from Sigma Aldrich (Germany). 2-(1H-benzotriazole-1-yl)-1,1,3,3-tetramethyluronium hexafluorophosphate (HBTU), N-hydroxybenzotriazole (HOBt), and N,N'-diisopropylcarbodiimide (DIC) were purchased from Biosolve (The Netherland). D_2O was from Armar GmbH (Germany).

Methods

Solid-phase peptide synthesis was carried out on an automatic peptide synthesizer (Syro I, Biotage). The analytical HPLC equipment was from Thermo Fisher Scientific (Ultimate 3000). The analytical column was from Thermo Fisher Scientific (Syncronis C_{18}, 4.6x250 mm). The gradient used for analytical HPLC was the following: 3% B for 8 min, up to 60% B over 35 min (A = H_2O with 0.06% TFA; B = CH_3CN with 0.05% TFA). MALDI-TOF mass spectra were recorded on an Autoflex mass spectrometer from Bruker Daltonics using α-cyano-4-hydroxycinnamic acid as matrix. The CD measurements were recorded on a Chirascan Plus CD spectrometer from Applied Photophysics. UV measurements were carried out on a Varian Cary UV-visible spectrophotometer.

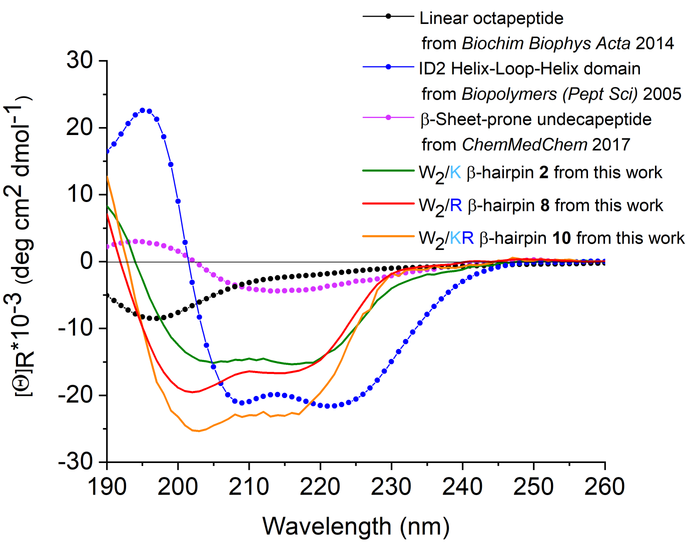

Figure S1. Comparison of the CD spectra of the β-hairpin peptides **2**, **8** and **10** with representative CD spectra for random-coil (black), β-sheet/hairpin (pink), and α-helical (blue) conformations. The black CD curve corresponds to the linear octapeptide Ac-MNGCYSRL-NH_2 that adopts a flexible (random coil) conformation (from Ref. [1]). The CD signature of a random coil conformation is characterized by weak intensity in the region 210-230 nm and a minimum below 200 nm. The pink CD curve arises from the linear undecapeptide Ac-KVEILQHVIDY-NH_2 adopting a β-sheet conformation upon self-aggregation (from the Supporting Information of Ref. [2]). The CD signature of a β-sheet conformation is characterized by a minimum close to 216 nm and a maximum close to 195 nm. Such CD signature is assigned also to β-hairpins, based on CD spectra of cyclic and linear model peptides for β-turns.[3] The blue CD curve corresponds the 41-residue-long peptide Ac-LYNMNDCYSKLKELVPSIPQNKKVSKMEILQHVIDYILDLQ-NH_2 reproducing the helix-loop-helix (HLH) domain of the ID2 protein (from Ref. [4]). The direct comparison shows that the CD spectrum of the β-hairpin peptides **2**, **8** and **10** is different from the CD spectrum of α-helical peptides, as the two minima and the positive contribution fall at significantly shorter wavelengths.

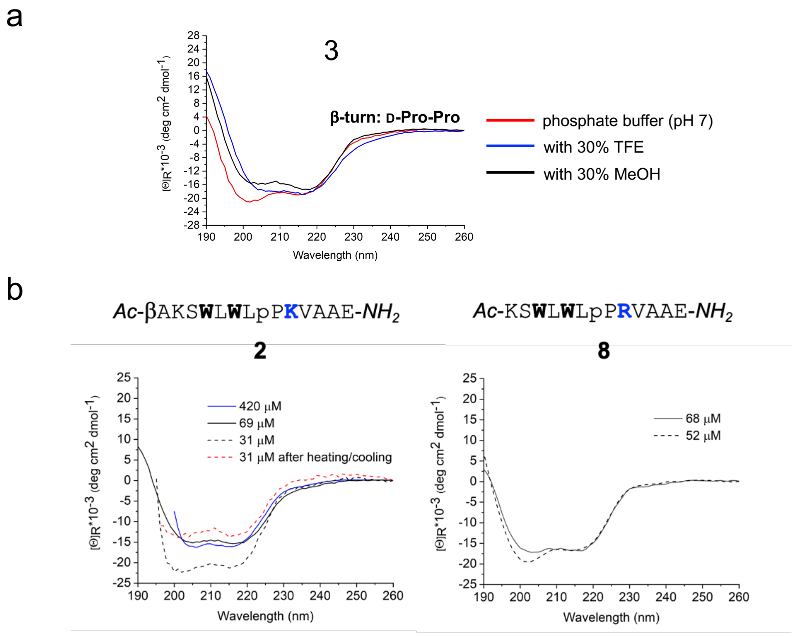

Figure S2. (a) CD spectra of peptide **3** at 56 µM. (b) CD spectra of peptides **2** and **8** at different concentrations.

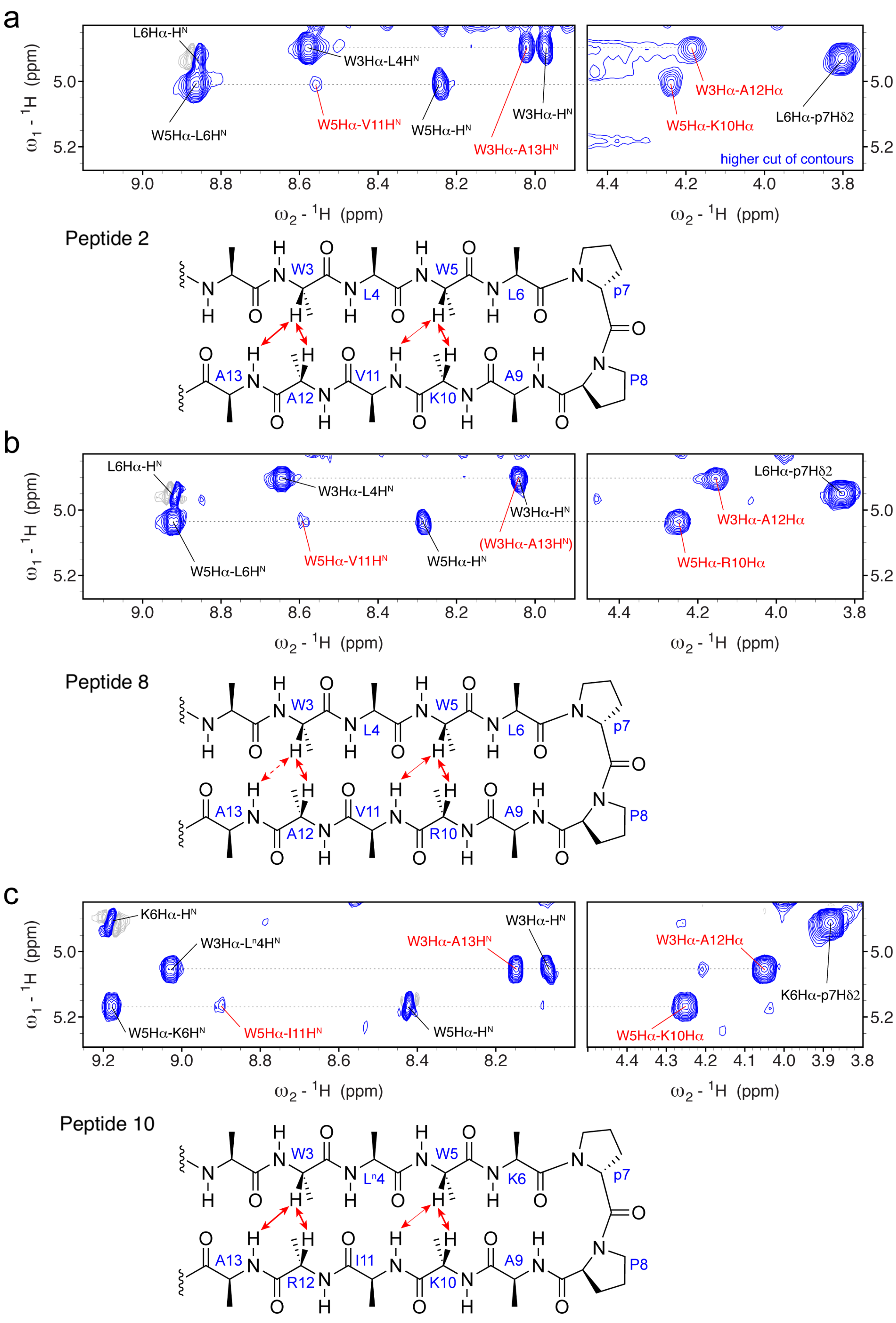

Figure S3. Backbone NOE contacts between cross-strand residues in peptides **2**, **8**, and **10** (top to bottom).

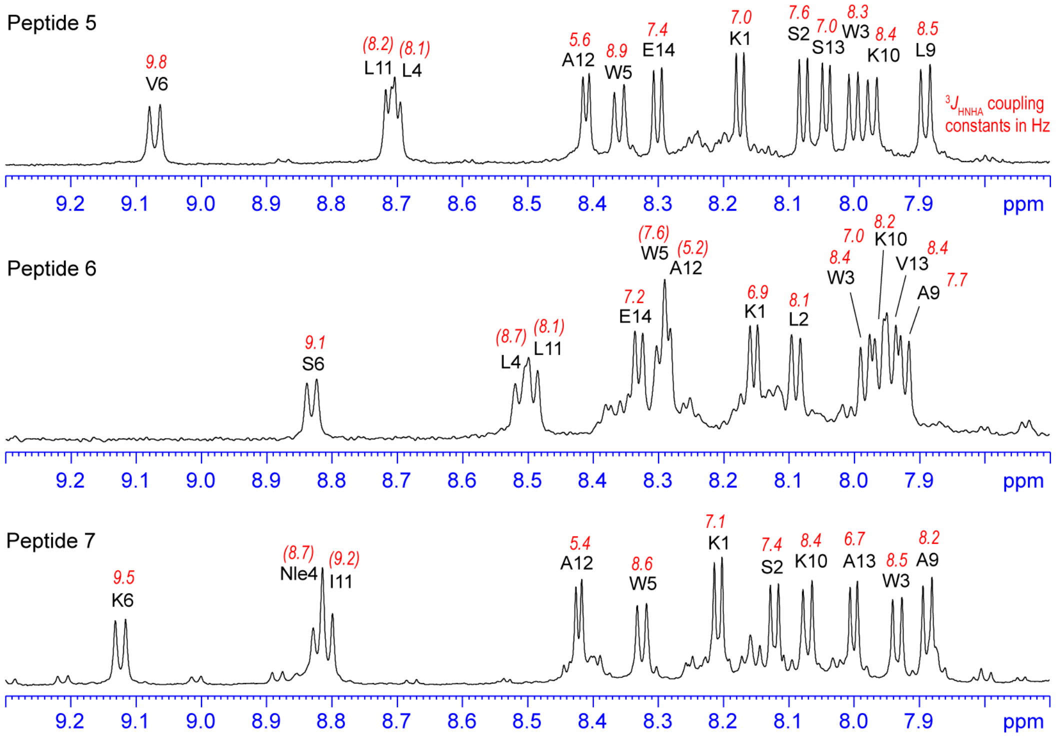

Figure S4. NMR spectra of peptides **5**, **6** and **7** measured in H_2O/D_2O at 298 K. Chemical shifts assignment and $^3J_{HNHA}$ scalar coupling constants are indicated. Values in brackets are estimates and could not be exactly extracted due to overlap.

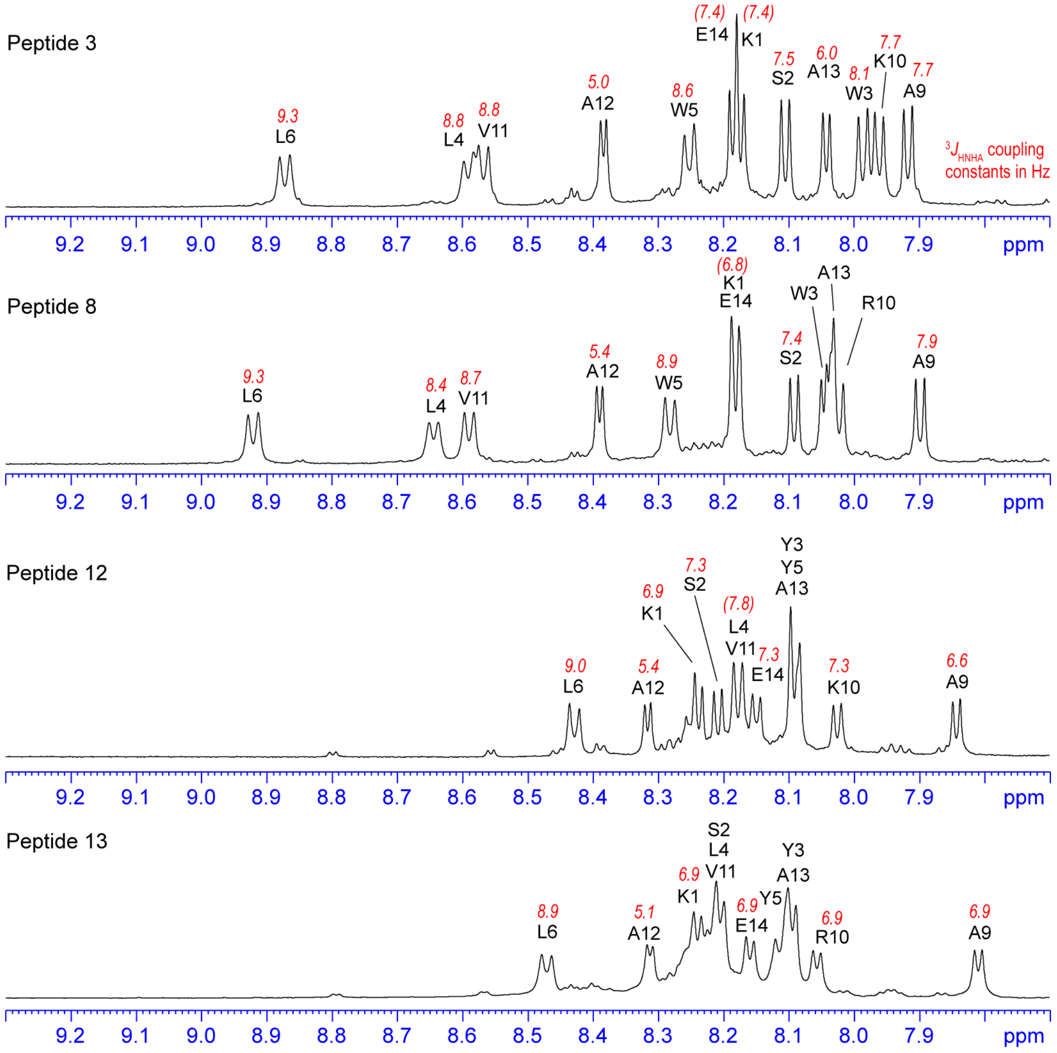

Figure S5. NMR spectra of peptides **3**, **8**, **12** and **13** measured in H_2O/D_2O at 298 K. Chemical shifts assignment and $^3J_{HNHA}$ scalar coupling constants are indicated. Values in brackets are estimates and could not be exactly extracted due to overlap.

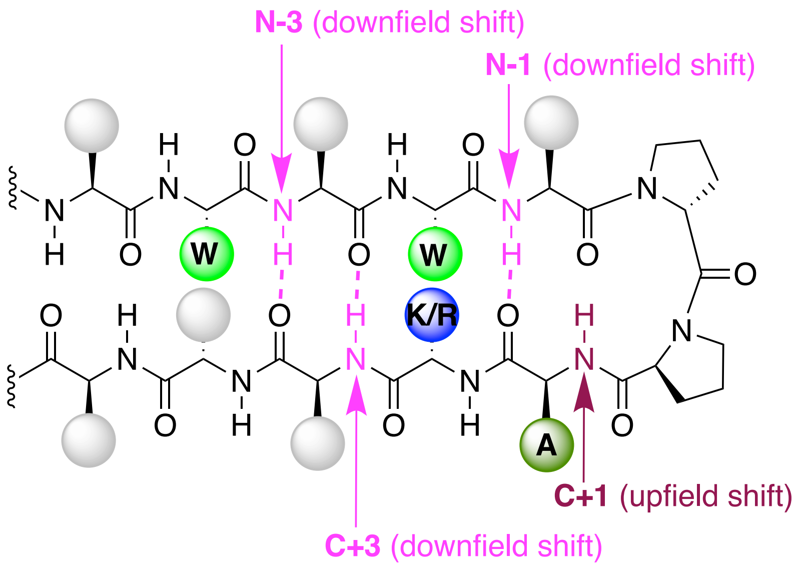

Figure S6. Scheme of the cross-strand H-bonds based on the chemical shifts deviations of the H^N protons (Figure 7b of this paper). Andersen and co-workers[5] identified the i/i+2 periodicity of downfield shifts for the H^N protons involved in cross-strand H-bonding: in detail, H-bonded H^N protons of the residues N-1, N-3, N-5… and C+3, C+5… display positive chemical shifts deviations (typically 0.8–1.2 ppm), whereas the C+1 H^N is shifted upfield. The latter upfield shift is due to the turn effect. In the case of our β-hairpin peptides, we also have downfield shifts (0.4–1 ppm) for the H^N protons of the residues N-1, N-3, and C+3, while the C+1 H^N is shifted upfield (-0.4 – -0.3 ppm), as shown in Figure 7b of this paper.

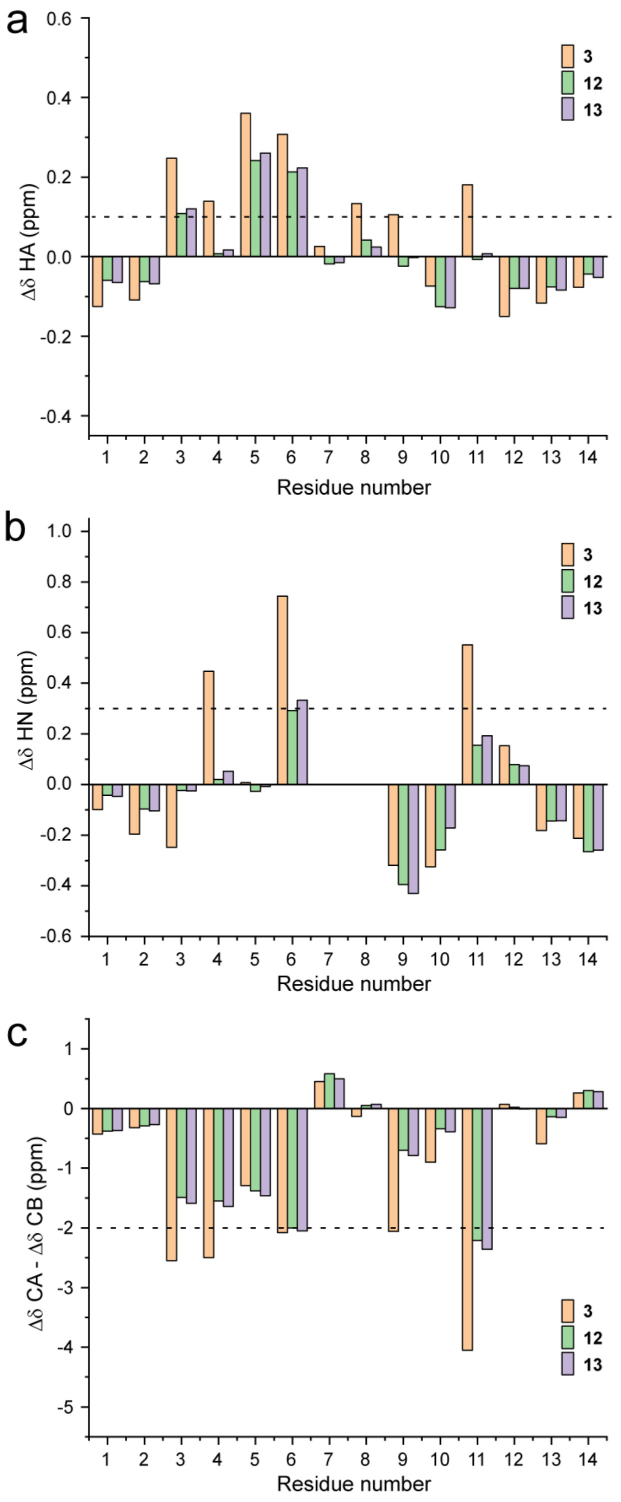

Figure S7. Backbone chemical shifts deviations of **3**, **12** and **13** from random-coil values[6]. (a) Hα chemical shifts deviations. (b) H^N chemical shifts deviations. (c) Chemical shifts deviations of Cα and Cβ. Here, [(Cα-Cα(r.c.))-(Cβ-Cβ(r.c.))] is plotted according to Marsh et al.[7]. No smoothening was applied.

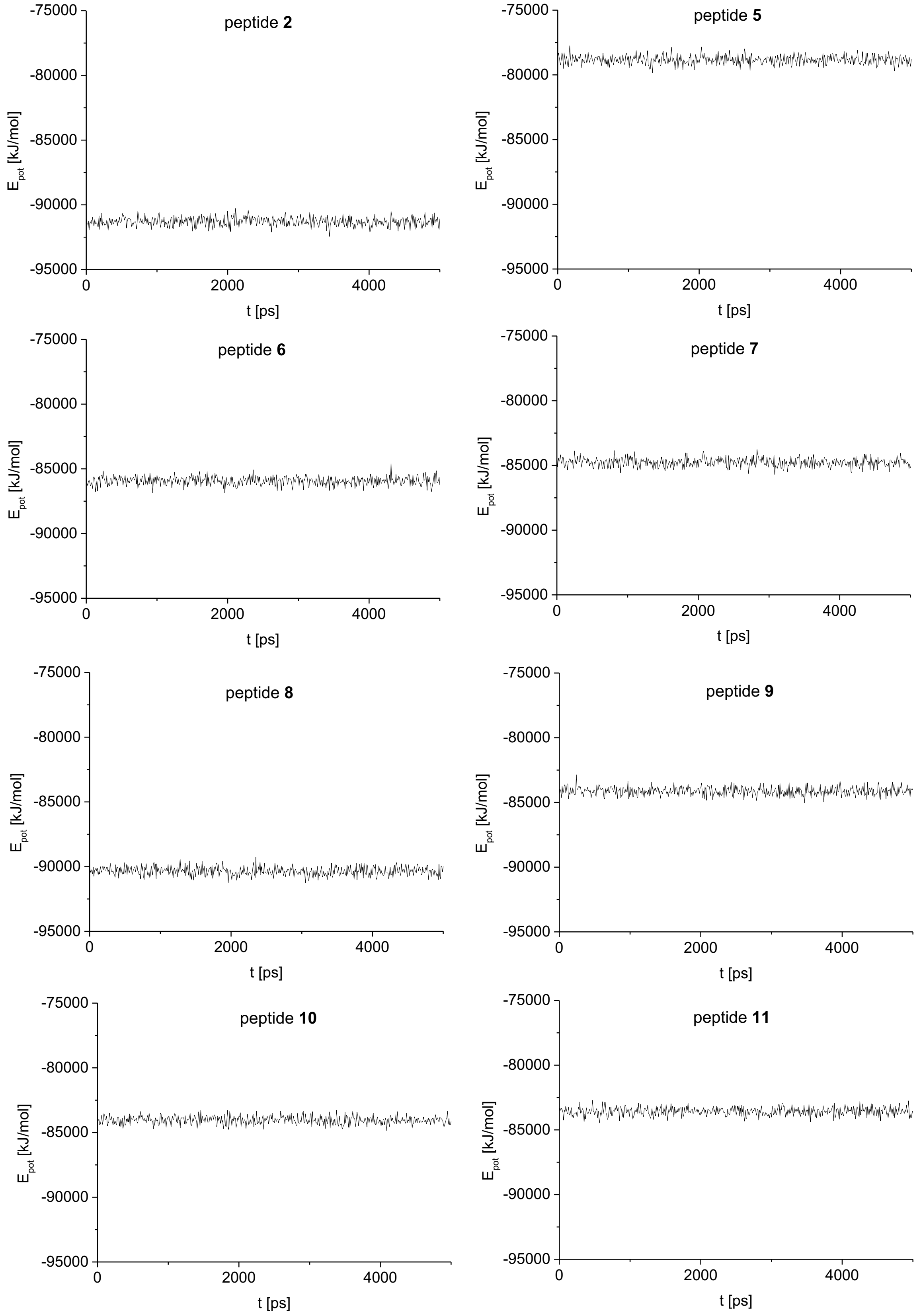

Figure S8. Potential energy of the NMR structure of peptides **2**, **5**-**11** during 5 ns production run.

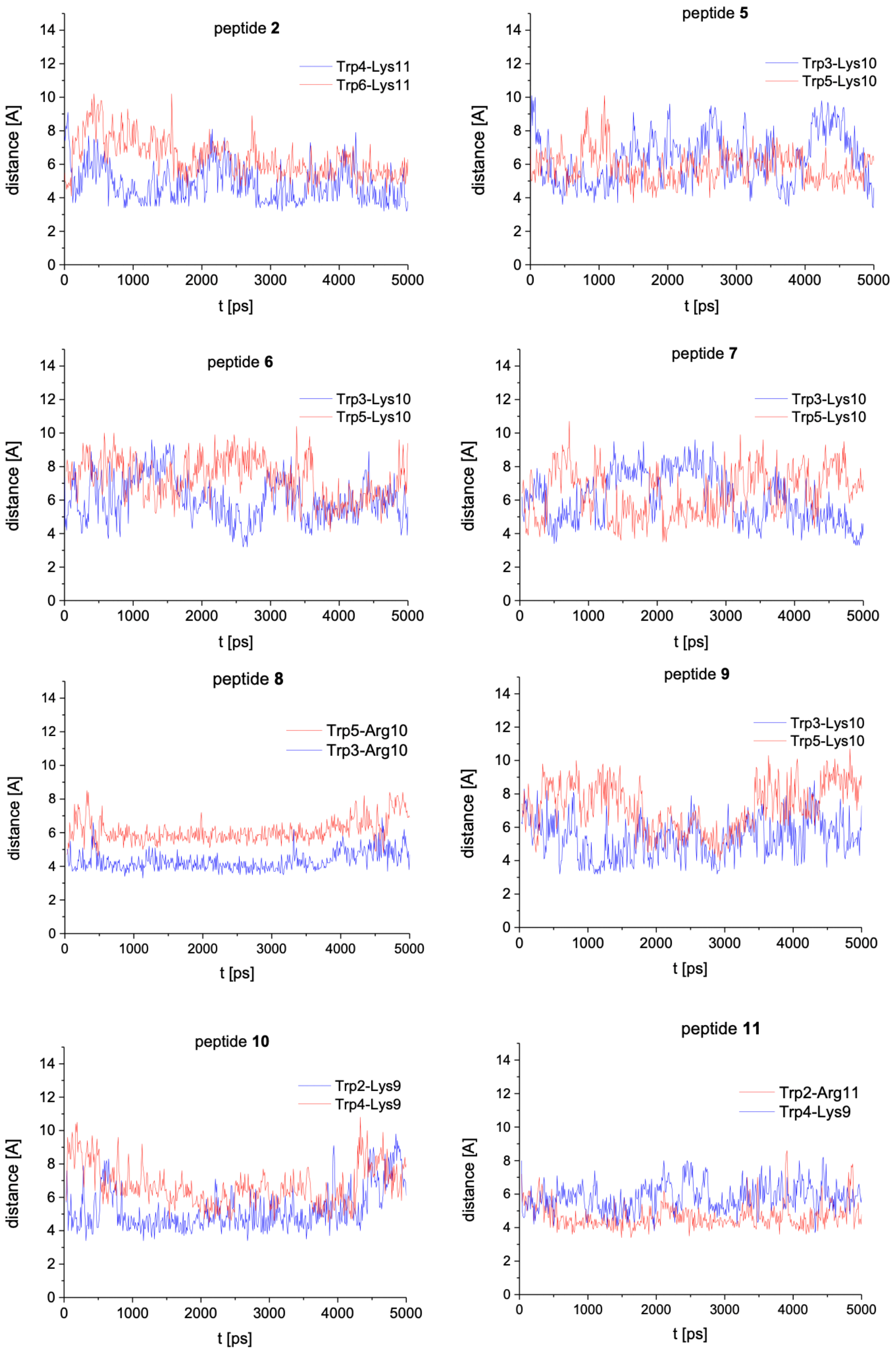

Figure S9. Distance between the center of the benzene ring of the indole of Trp residues at N-2 and N-4 and the Nζ/Nε of Lys/Arg at positions C+2 and C+4 during 5 ns long MD simulation (for Arg, same results were obtained by considering the center of the guanidinium group). The residue number in the legends is based on the amino-acid sequence of each peptide.

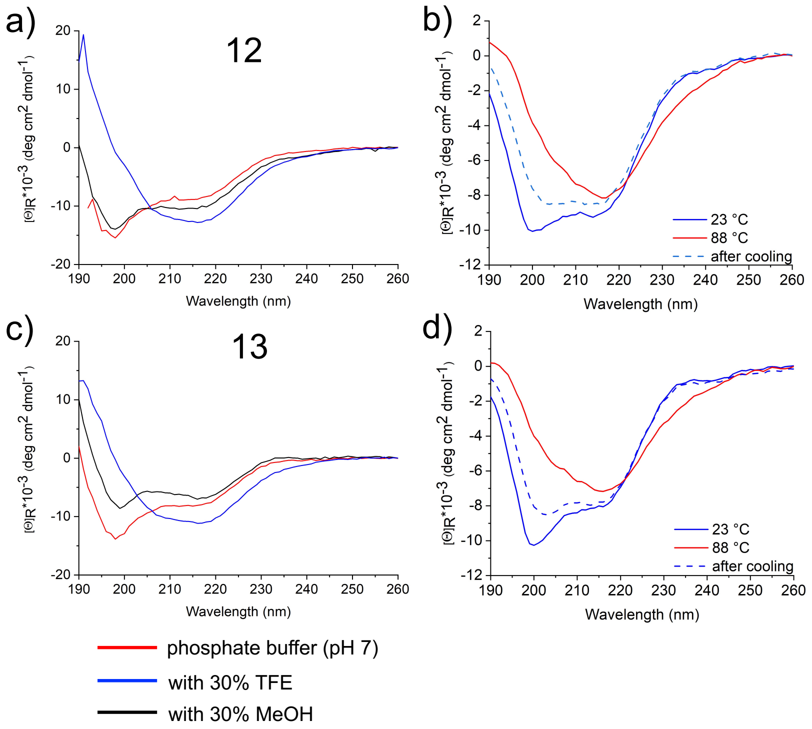

Figure S10. CD spectra of peptides **12** (a and b) and **13** (c and d). Peptide concentrations: 88 μM (a), 157 μM (b), 70 μM (c), 162 μM (d). No significant differences were observed between the peptides containing Lys (**12**) or Arg (**13**). Moreover, the temperature dependence of the two CD minima was anomalous: indeed, the minimum at 216 nm remained nearly constant during the whole thermal transition (heating and cooling), whereas the minimum near 200 nm disappeared during the heating phase and reappeared, though red-shifted, during the cooling phase. Since both peptides are not well-structured, the temperature-induced CD changes are likely to reflect changes in the contribution of the aromatic side chains rather than of the backbone. This also suggests that the minimum at the shorter wavelength is likely to contain a significant contribution of the aromatic residues.[8]

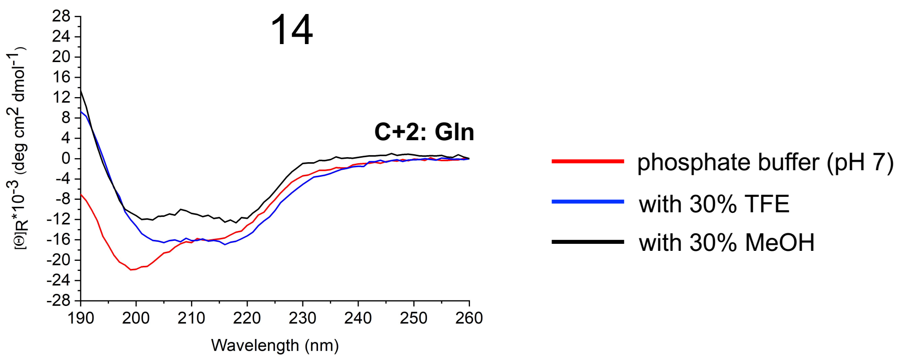

Figure S11. CD spectra of peptide **14** at 35 μM.

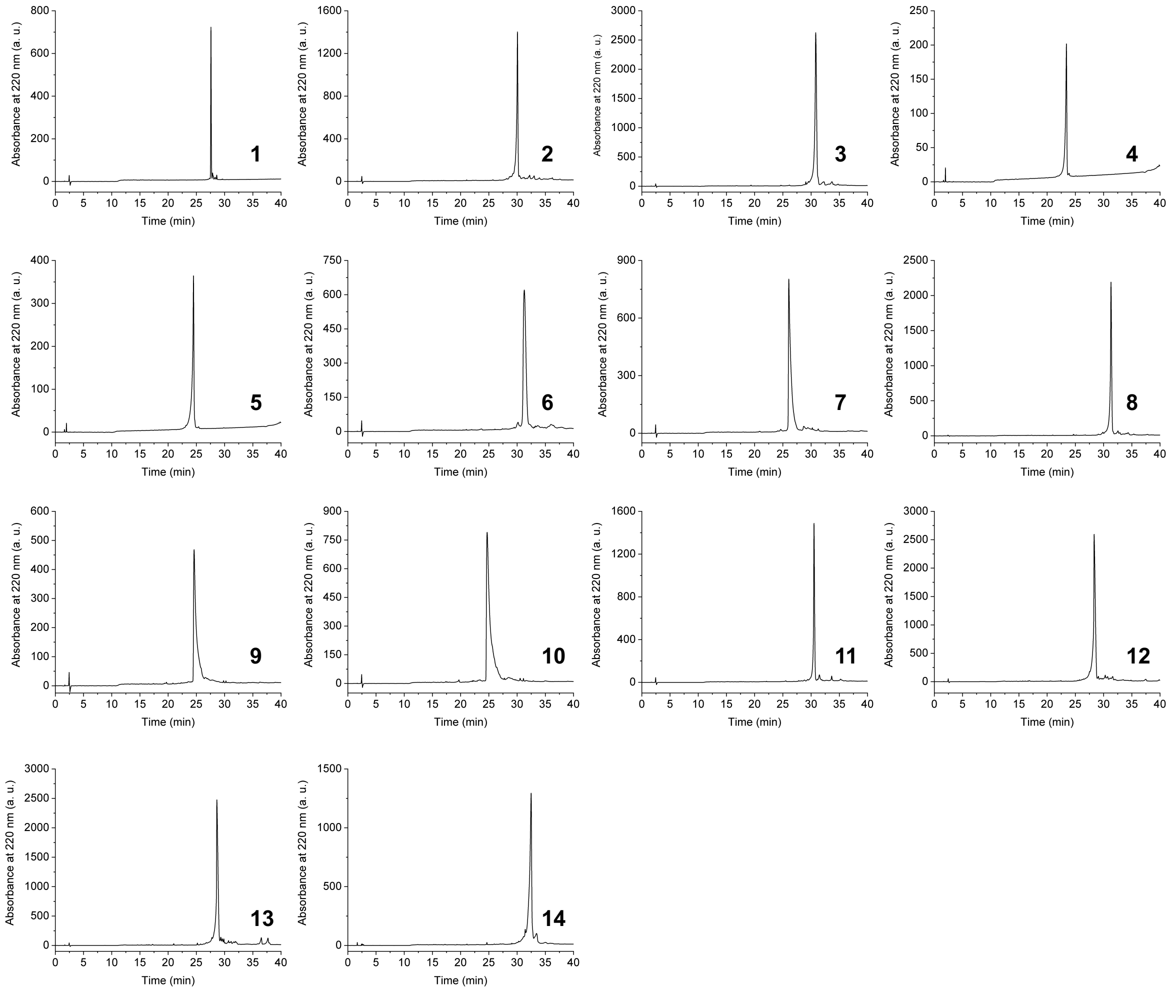

Figure S12. Analytical HPLC of the synthetic peptides used in this work (see Table S16 for t_R values and gradient).

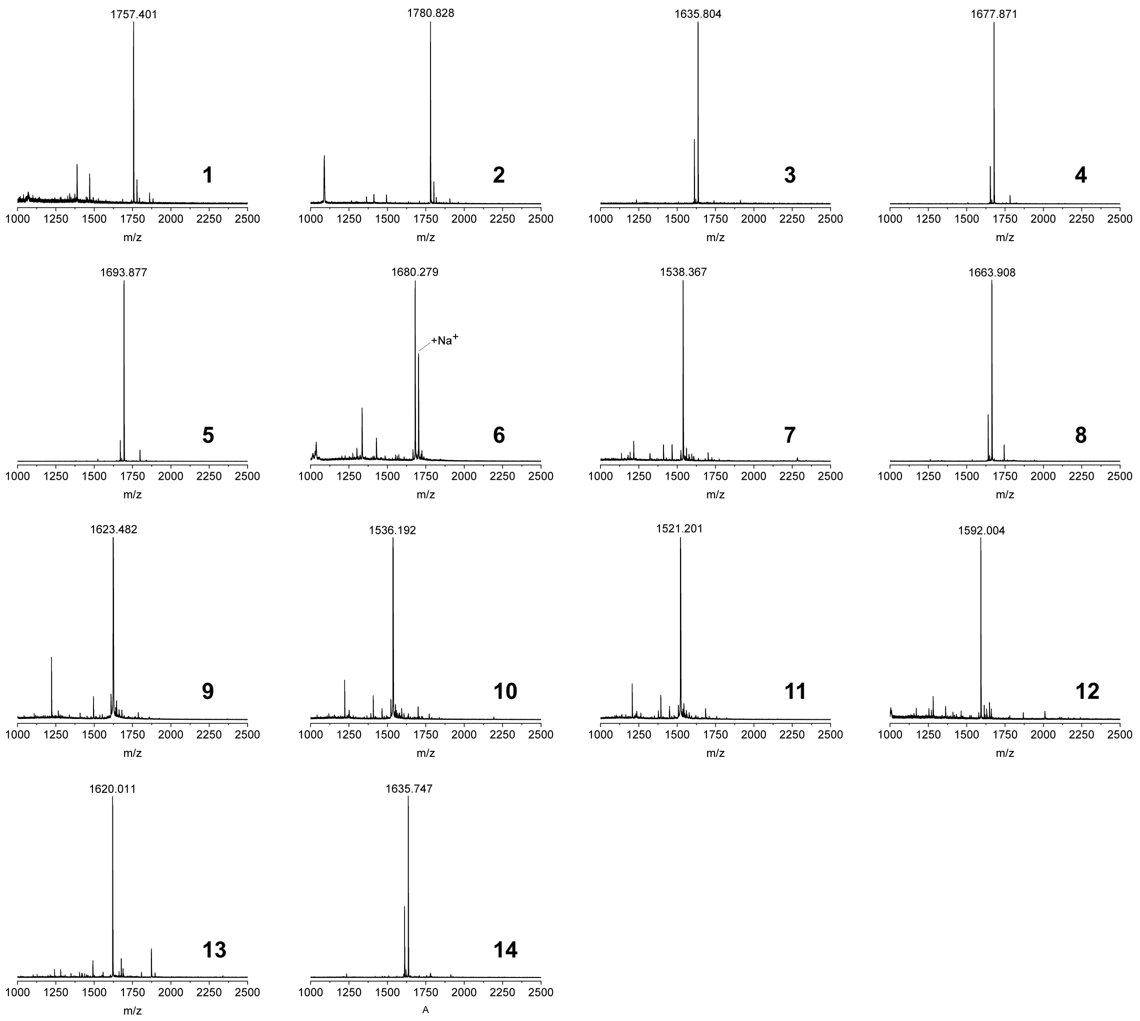

Figure S13. MALDI-TOF-MS of the synthetic peptides used in this work (see Table S16 for M_{theor}).

Table S1. NOE contacts between Lys/Arg at position C+2 or C+4 and Trp at position N-2 or N-4 in peptides **2**, **5**-**11** in water.

Peptide **2**	Peptide **5**	Peptide **6**
4 TRP QB 11 LYS HB2<br>4 TRP QB 11 LYS HB3<br>4 TRP QB 11 LYS HG3<br>4 TRP QB 11 LYS HD2<br>4 TRP QB 11 LYS HD3<br>4 TRP HE3 11 LYS QG<br>4 TRP HE3 11 LYS HB2<br>4 TRP HE3 11 LYS HB3<br><br>6 TRP HA 11 LYS HA<br>6 TRP HD1 11 LYS HA<br>6 TRP HE3 11 LYS HA<br>6 TRP HH2 11 LYS HD3<br>6 TRP HH2 11 LYS QE<br>6 TRP HZ2 11 LYS HD2<br>6 TRP HZ2 11 LYS HD3<br>6 TRP HZ2 11 LYS QE<br>6 TRP HZ3 11 LYS HA<br>6 TRP HZ3 11 LYS HB3	4 TRP HD1 11 LYS HG2<br>4 TRP HD1 11 LYS QE<br>4 TRP HE3 11 LYS HB2<br>4 TRP HE3 11 LYS HB3<br>4 TRP HE3 11 LYS HG2<br>4 TRP HE3 11 LYS HG3<br>4 TRP HE3 11 LYS QE<br>4 TRP HZ3 11 LYS QE<br><br>6 TRP HA 11 LYS HA<br>6 TRP HA 11 LYS HA<br>6 TRP HD1 11 LYS<br>6 TRP HE3 11 LYS HA<br>6 TRP HE3 11 LYS HB3<br>6 TRP HZ3 11 LYS HB3<br>6 TRP HH2 11 LYS HB3<br>6 TRP HH2 11 LYS QD<br>6 TRP HH2 11 LYS QE<br>6 TRP HZ2 11 LYS HB3<br>6 TRP HZ2 11 LYS HG3<br>6 TRP HZ2 11 LYS QD<br>6 TRP HZ2 11 LYS QE	4 TRP QB 11 LYS QG<br>4 TRP HD1 11 LYS HB2<br>4 TRP HE3 11 LYS QG<br>4 TRP HE3 11 LYS QE<br>4 TRP HZ2 11 LYS QE<br><br>6 TRP HH2 11 LYS QD<br>6 TRP HA 11 LYS HA<br>6 TRP HE3 11 LYS HA<br>6 TRP HZ3 11 LYS QG<br>6 TRP HE3 11 LYS QG<br>6 TRP HH2 11 LYS QD<br>6 TRP HZ2 11 LYS QD
Peptide **7**	Peptide **8**	Peptide **9**
4 TRP QB 11 LYS HG2<br>4 TRP QB 11 LYS HG3<br>4 TRP QB 11 LYS HD3<br>4 TRP QB 11 LYS HB2<br>4 TRP HD1 11 LYS HG2<br>4 TRP HD1 11 LYS HG3<br>4 TRP HD1 11 LYS HE2<br>4 TRP HD1 11 LYS HE3<br>4 TRP HE3 11 LYS HB3<br><br>4 TRP HE3 11 LYS HG2<br>4 TRP HE3 11 LYS HG3<br>6 TRP HA 11 LYS HA<br>6 TRP HZ3 11 LYS H<br>6 TRP HE3 11 LYS HA<br>6 TRP HH2 11 LYS HB3<br>6 TRP HZ3 11 LYS HB3<br>6 TRP HH2 11 LYS HD2<br>6 TRP HZ2 11 LYS HD2<br>6 TRP HH2 11 LYS HD3<br>6 TRP HZ2 11 LYS HD3<br>6 TRP HZ3 11 LYS HG2<br>6 TRP HZ3 11 LYS HG3	4 TRP QB 11 ARG HB2<br>4 TRP QB 11 ARG HB3<br>4 TRP QB 11 ARG QG<br>4 TRP QB 11 ARG HD3<br>4 TRP QB 11 ARG HE<br>4 TRP HE3 11 ARG HA<br>4 TRP HE3 11 ARG HB2<br>4 TRP HE3 11 ARG HB3<br>4 TRP HE3 11 ARG QG<br>4 TRP HZ3 11 ARG QG<br><br>6 TRP HA 11 ARG HA<br>6 TRP HE3 11 ARG HA<br>6 TRP HE3 11 ARG HB3<br>6 TRP HE3 11 ARG QG<br>6 TRP HZ3 11 ARG HB3<br>6 TRP HZ3 11 ARG QG<br>6 TRP HH2 11 ARG HB3<br>6 TRP HH2 11 ARG QG<br>6 TRP HH2 11 ARG HD2<br>6 TRP HH2 11 ARG HD3<br>6 TRP HZ2 11 ARG HD2<br>6 TRP HZ2 11 ARG HD3	4 TRP QB 11 LYS QG<br>4 TRP QB 11 LYS HD3<br>4 TRP QB 11 LYS HB2<br>4 TRP HD1 11 LYS QG<br>4 TRP HD1 11 LYS HE2<br>4 TRP HD1 11 LYS HE3<br>4 TRP HE3 11 LYS HB2<br>4 TRP HE3 11 LYS HB3<br>4 TRP HE3 11 LYS QG<br>4 TRP HZ3 11 LYS QG<br><br>6 TRP HA 11 LYS HA<br>6 TRP HE3 11 LYS HA<br>6 TRP HD1 11 LYS HA<br>6 TRP HH2 11 LYS HB3<br>6 TRP HE3 11 LYS HB3<br>6 TRP HH2 11 LYS HD2<br>6 TRP HZ2 11 LYS HD2<br>6 TRP HH2 11 LYS HD3<br>6 TRP HZ2 11 LYS HD3<br>6 TRP HZ2 11 LYS QG<br>6 TRP HH2 11 LYS QG<br>6 TRP HZ3 11 LYS QG<br>6 TRP HE3 11 LYS QG<br>6 TRP HD1 11 LYS QG<br><br>4 TRP HA 13 ARG HA<br>4 TRP QB 13 ARG HA<br>4 TRP HZ3 13 ARG H<br>4 TRP HE3 13 ARG HA<br>4 TRP HZ3 13 ARG HA<br>4 TRP HZ3 13 ARG HB3<br>4 TRP HZ3 13 ARG HG2<br>4 TRP HZ3 13 ARG HG3<br>4 TRP HZ3 13 ARG QD

Table S1. NOE contacts between Lys/Arg at position C+2 or C+4 and Trp at position N-2 or N-4 in peptides **2**, **5**-**11** in water (continued).

Peptide **10**	Peptide **11**
4 TRP HB3 11 LYS HG2	4 TRP HB3 11 LYS HG2
4 TRP HB3 11 LYS HG3	4 TRP HB3 11 LYS HG3
4 TRP HB3 11 LYS HD2	4 TRP HB3 11 LYS QD
4 TRP HD1 11 LYS HE2	4 TRP HZ3 11 LYS QD
4 TRP HD1 11 LYS HE3	4 TRP HE3 11 LYS HB2
4 TRP HE3 11 LYS HA	4 TRP HE3 11 LYS HG2
4 TRP HE3 11 LYS HB2	4 TRP HE3 11 LYS HG3
4 TRP HE3 11 LYS HB3	4 TRP HE3 11 LYS QD
4 TRP HE3 11 LYS HG2	
4 TRP HE3 11 LYS HG3	6 TRP HZ3 11 LYS H
	6 TRP HA 11 LYS HA
6 TRP HA 11 LYS HA	6 TRP HE3 11 LYS HA
6 TRP HE3 11 LYS HA	6 TRP HZ2 11 LYS HG3
6 TRP HZ2 11 LYS HB3	6 TRP HZ3 11 LYS HG2
6 TRP HH2 11 LYS HB3	6 TRP HZ3 11 LYS HG3
6 TRP HZ3 11 LYS HB3	6 TRP HE3 11 LYS HG2
6 TRP HE3 11 LYS HB3	6 TRP HE3 11 LYS HG3
6 TRP HZ2 11 LYS HG3	6 TRP HZ2 11 LYS QD
6 TRP HZ3 11 LYS HG3	6 TRP HH2 11 LYS QD
6 TRP HE3 11 LYS HG3	6 TRP HZ3 11 LYS QD
6 TRP HZ2 11 LYS HD3	6 TRP HE3 11 LYS QD
6 TRP HZ3 11 LYS HD3	6 TRP HZ2 11 LYS QE
6 TRP HZ2 11 LYS HE2	
6 TRP HZ2 11 LYS HE3	4 TRP HA 13 ARG HA
	4 TRP HE3 13 ARG H
4 TRP HA 13 ARG HA	4 TRP HZ3 13 ARG H
4 TRP HE3 13 ARG H	4 TRP HE3 13 ARG HA
4 TRP HZ3 13 ARG H	4 TRP HZ3 13 ARG HA
4 TRP HE3 13 ARG HA	4 TRP HZ3 13 ARG HB3
4 TRP HZ3 13 ARG HA	4 TRP HZ3 13 ARG HG2
4 TRP HZ3 13 ARG HB3	4 TRP HZ3 13 ARG HG3
4 TRP HZ3 13 ARG HG2	4 TRP HH2 13 ARG HB3
4 TRP HZ3 13 ARG HG3	4 TRP HZ2 13 ARG QE
4 TRP HZ2 13 ARG QE	

Table S2. Chemical shifts of peptide **1** in water.

Residue	H^N	N	H^α	C^α	H^β	C^β	Others
**Ac -1**			1.921	24.6			
**βA 0**	7.894		2.427<br>2.467	37.7	3.354	38.5	
**K 1**	8.249		4.172	56.3	1.574	33.0	Hγ 1.278, 1.321, C^γ 24.8; H^δ 1.571, C^δ 29.0; H^ε 2.874, C^ε 41.9; H^ζ 7.489
**S 2**	8.172		4.348	58.2	3.757<br>3.799	63.7	
**W 3**	8.040		4.746	56.9	3.089	30.1	$H^{\delta1}$ 7.116, $C^{\delta1}$ 127.1; $H^{\varepsilon1}$ 10.080; $H^{\varepsilon3}$ 7.458, $C^{\varepsilon3}$ 120.8; $H^{\zeta2}$ 7.471, $C^{\zeta2}$ 114.6; $H^{\zeta3}$ 7.017, $C^{\zeta3}$ 122.1; $H^{\eta2}$ 7.216, $C^{\eta2}$ 124.7
**L 4**	8.239		4.298	55.1	1.429	43.2	H^γ 1.361, C^γ 26.8; Hδ1 0.803, Cδ1 23.8; Hδ2 0.834, Cδ2 24.9
**W 5**	8.068		4.818	56.9	3.152<br>3.241	29.5	$H^{\delta1}$ 7.205, $C^{\delta1}$ 126.9; $H^{\varepsilon1}$ 10.130, $N^{\varepsilon1}$; $H^{\varepsilon3}$ 7.466, $C^{\varepsilon3}$ 120.7; $H^{\zeta2}$ 7.421, $C^{\zeta2}$ 114.6; $H^{\zeta3}$ 7.091, $C^{\zeta3}$ 122.1; $H^{\eta2}$ 7.175, $C^{\eta2}$ 124.4
**L 6**	8.328		4.366	55.0	1.482<br>1.545	43.3	H^γ 1.430, C^γ 26.9; Hδ1 0.843, $C^{\delta1}$ 24.6; $H^{\delta2}$ 0.882, $C^{\delta2}$ 24.0
**N 7**	8.644		4.488	53.8	2.646<br>2.868	38.2	H^δ 6.895, 7.532
**G 8**	8.331			45.5	3.784<br>3.996		
**A 9**	7.886		4.400	51.7	1.346	20.1	
**K 10**	8.169		4.239	56.1	1.426<br>1.534	32.8	H^γ 0.995, 1.086, C^γ 24.7; H^δ 1.325, C^δ 28.9; H^ε 2.629, 2.599, C^ε 41.5; H^ζ 7.303
**V 11**	8.363		4.189	61.5	2.030	33.7	Hγ1 0.913, Cγ1 21.2; Hγ2 0.887, Cγ2 20.6
**A 12**	8.384		4.239	52.4	1.306	19.2	
**A 13**	8.149		4.247	52.3	1.311	19.2	
**E 14**	8.224		4.223	56.2	1.924<br>2.036	29.5	H^γ 2.360, C^γ 34.0
**βA 15**	8.068		2.472	37.3	3.417<br>3.485	38.5	
**amide 16**	6.856<br>7.550						

Table S3. Chemical shifts of peptide **2** in water.

Residue	H^N	N	H^α	C^α	H^β	C^β	Others
**Ac -1**			1.924	24.6			
**βA 0**	7.903		2.441<br>2.476	37.7	3.367	38.5	
**K 1**	8.242		4.202	56.2	1.578	33.0	H^γ 1.292, 1.329, C^γ 24.8; H^δ 1.585, C^δ 29.0; H^ε 2.889, C^ε 41.9; H^ζ 7.496
**S 2**	8.136		4.369	58.1	3.730	63.8	
**W 3**	7.974		4.904	56.3	2.944	31.0	$H^{\delta1}$ 7.041, $C^{\delta1}$ 127.3; $H^{\varepsilon1}$ 9.982; $H^{\varepsilon3}$ 7.308, $C^{\varepsilon3}$ 120.7; $H^{\zeta2}$ 7.444, $C^{\zeta2}$ 114.5; $H^{\zeta3}$ 6.807, $C^{\zeta3}$ 121.9; $H^{\eta2}$ 7.178, $C^{\eta2}$ 124.6
**L 4**	8.577		4.471	54.3	1.466	43.8	H^γ 1.389, C^γ 26.9; $H^{\delta1}$ 0.864, $C^{\delta1}$ 25.0; $H^{\delta2}$ 0.836, $C^{\delta2}$ 24.1
**W 5**	8.240		5.018	56.8	3.053<br>3.335	30.3	$H^{\delta1}$ 7.199, $C^{\delta1}$ 127.0; $H^{\varepsilon1}$ 10.050; $H^{\varepsilon3}$ 7.610, $C^{\varepsilon3}$ 121.6; $H^{\zeta2}$ 7.412, $C^{\zeta2}$ 114.1; $H^{\zeta3}$ 7.028, $C^{\zeta3}$ 122.1; $H^{\eta2}$ 7.140, $C^{\eta2}$ 124.4
**L 6**	8.866		4.938	52.0	1.523<br>1.626	42.7	H^γ 1.542, C^γ 27.1; $H^{\delta1}$ 0.927, $C^{\delta1}$ 25.0; $H^{\delta2}$ 0.911, $C^{\delta2}$ 24.2
**p 7**	-		4.768	61.5	1.922<br>2.305	30.5	H^γ 2.036, 2.147, C^γ 27.7; H^δ 3.674, 3.812, C^δ 50.9
**P 8**	-		4.558	63.6	2.132<br>2.264	32.2	H^γ 2.015, 2.075, C^γ 26.5; H^δ 3.723, 3.974, C^δ 50.3
**A 9**	7.920		4.422	51.8	1.410	20.3	
**K 10**	7.967		4.240	55.7	1.010<br>1.175	33.0	H^γ 0.575, C^γ 24.4; H^δ 0.872, 0.916, C^δ 28.9; H^ε 2.176, C^ε 41.3; H^ζ 7.066
**V 11**	8.557		4.289	61.1	2.035	34.3	$H^{\gamma1}$ 0.896, $C^{\gamma1}$ 21.2; $H^{\gamma2}$ 0.847, $C^{\gamma2}$ 20.4
**A 12**	8.376		4.187	52.3	1.234	19.2	
**A 13**	8.023		4.205	52.2	1.236	19.0	
**E 14**	8.165		4.249	55.7	1.925<br>2.045	29.0	H^γ 2.417, C^γ 32.6
**βA 15**	8.058		2.465	37.2	3.406<br>3.480	38.5	
**amide 16**	6.848<br>7.541						

Table S4. Chemical shifts of peptide **3** in water.

Residue	H^N	N	$H^α$	$C^α$	$H^β$	$C^β$	Others
**Ac 0**			2.005	24.4			
**K 1**	8.191		4.195	56.2	1.566	33.1	$H^γ$ 1.278, 1.324, $C^γ$ 24.7; $H^δ$ 1.568, $C^δ$ 29.0; $H^ε$ 2.880, $C^ε$ 41.9; $H^ζ$ 7.492
**S 2**	8.114		4.361	58.1	3.706	63.8	
**W 3**	8.002		4.907	56.3	2.930	31.0	$H^{δ1}$ 7.062, $C^{δ1}$ 127.4; $H^{ε1}$ 9.987; $H^{ε3}$ 7.300, $C^{ε3}$ 120.7; $H^{ζ2}$ 7.438, $C^{ζ2}$ 114.5; $H^{ζ3}$ 6.820, $C^{ζ3}$ 121.9; $H^{η2}$ 7.171, $C^{η2}$ 124.6
**L 4**	8.607		4.479	54.3	1.462	43.8	$H^γ$ 1.379, $C^γ$ 26.9; $Hδ^1$ 0.827, $C^{δ1}$ 24.1; $H^{δ2}$ 0.847, $C^{δ2}$ 25.0
**W 5**	8.258		5.020	56.8	3.046<br>3.329	30.3	$H^{δ1}$ 7.202, $C^{δ1}$ 127.0; $H^{ε1}$ 10.050; $H^{ε3}$ 7.609, $C^{ε3}$ 121.5; $H^{ζ2}$ 7.392, $C^{ζ2}$ 114.1; $H^{ζ3}$ 7.013, $C^{ζ3}$ 122.0; $H^{η2}$ 7.122, $C^{η2}$ 124.4
**L 6**	8.884		4.937	52.0	1.524<br>1.622	42.7	$H^γ$ 1.527, $C^γ$ 27.0; $H^{δ1}$ 0.909, $C^{δ1}$ 24.2; $H^{δ2}$ 0.907, $C^{δ2}$ 25.0
**p 7**			4.756	61.5	1.904<br>2.296	30.5	$H^γ$ 2.018, 2.133, $C^γ$ 27.6; $H^δ$ 3.667, 3.819, $C^δ$ 50.9
**P 8**			4.553	63.6	2.129<br>2.249	32.2	$H^γ$ 2.000, 2.055, $C^γ$ 26.5; $H^δ$ 3.717, 3.973, $C^δ$ 50.3
**A 9**	7.921		4.425	51.8	1.410	20.4	
**K 10**	7.965		4.246	55.6	0.989<br>1.141	33.0	$H^γ$ 0.557, $C^γ$ 24.4; $H^δ$ 0.860, $C^δ$ 28.9; $H^ε$ 2.096, 2.142, $C^ε$ 41.2
**V 11**	8.582		4.301	61.1	2.030	34.3	$H^{γ1}$ 0.893, $C^{γ1}$ 21.2; $H^{γ2}$ 0.840, $C^{γ2}$ 20.3,
**A 12**	8.393		4.170	52.4	1.223	18.9	
**A 13**	8.058		4.203	52.2	1.230	19.3	
**E 14**	8.207		4.273	55.4	1.936<br>2.088	28.9	$H^γ$ 2.402, $C^γ$ 32.8
**amide 15**	7.082<br>7.524						

Table S5. Chemical shifts of peptide **5** in water.

Residue	H^N	N	H^α	C^α	H^β	C^β	Others
**Ac 0**			2.003	24.4			
**K 1**	8.177	126.4	4.210	56.2	1.572	33.1	H^γ 1.283, 1.337, C^γ 24.8; H^δ 1.577, C^δ 29.0; H^ε 2.872, C^ε 41.9; H^ζ 7.485
**S 2**	8.079	116.3	4.388	57.9	3.712	63.9	
**W 3**	8.002	121.7	4.961	56.2	2.893	31.2	$H^{\delta1}$ 7.079, $C^{\delta1}$ 127.3; $H^{\varepsilon1}$ 10.010, $N^{\varepsilon1}$ 129.2; $H^{\varepsilon3}$ 7.318, $C^{\varepsilon3}$ 120.8; $H^{\zeta2}$ 7.462, $C^{\zeta2}$ 114.4; $H^{\zeta3}$ 6.894, $C^{\zeta3}$ 121.9; $H^{\eta2}$ 7.193, $C^{\eta2}$ 124.6
**L 4**	8.704	123.0	4.571	54.3	1.520	44.2	H^γ 1.427, C^γ 26.9; $H^{\delta1}$ 0.872, $C^{\delta1}$ 25.0; $H^{\delta2}$ 0.828, $C^{\delta2}$ 24.4
**W 5**	8.360	122.2	5.204	56.7	3.020<br>3.330	30.5	$H^{\delta1}$ 7.207, $C^{\delta1}$ 126.9; $H^{\varepsilon1}$ 10.050, $N^{\varepsilon1}$ 128.4; $H^{\varepsilon3}$ 7.604, $C^{\varepsilon3}$ 121.6; $H^{\zeta2}$ 7.384, $C^{\zeta2}$ 114.0; $H^{\zeta3}$ 7.006, $C^{\zeta3}$ 122.0; $H^{\eta2}$ 7.134, $C^{\eta2}$ 124.3
**V 6**	9.073	124.9	4.591	59.5	2.115	32.4	$H^{\gamma1}$ 0.951, $C^{\gamma1}$ 21.1; $H^{\gamma2}$ 0.930, $C^{\gamma2}$ 20.5
**p 7**			4.804	61.4	1.917<br>2.331	30.4	H^γ 2.048, 2.165, C^γ 27.7; H^δ 3.664, 3.925, C^δ 51.1
**P 8**			4.634	63.6	2.175<br>2.271	32.3	H^γ 1.953, 2.106, C^γ 26.5; H^δ 3.747, 3.999, C^δ 50.2
**L 9**	7.892	122.4	4.433	54.5	1.635<br>1.760	43.4	H^γ 1.539, C^γ 27.1; $H^{\delta1}$ 0.927, $C^{\delta1}$ 24.7; $H^{\delta2}$ 0.857, $C^{\delta2}$ 23.8
**K 10**	7.973	124.3	4.067	55.2	0.622<br>1.050	32.6	H^γ 0.253, 0.433, C^γ 24.3; H^δ 0.804, C^δ 28.8; H^ε 1.992, C^ε 41.0; H^ζ 6.959
**L 11**	8.714	126.9	4.541	54.1	1.554	44.1	H^γ 1.510, C^γ 27.1; $H^{\delta1}$ 0.875, $C^{\delta1}$ 24.9; $H^{\delta2}$ 0.845, $C^{\delta2}$ 24.4
**A 12**	8.411	125.0	4.246	52.3	1.216	19.0	
**S 13**	8.043	115.2	4.338	57.7	3.638<br>3.735	63.8	
**E 14**	8.301	122.4	4.345	55.4	1.939<br>2.135	28.8	H^γ 2.436, C^γ 32.8
**amide 15**	7.109<br>7.542	107.8					

Table S6. Chemical shifts of peptide **6** in water.

Residue	H^N	N	H^α	C^α	H^β	C^β	Others
**Ac 0**			2.002	24.4			
**K 1**	8.156	126.6	4.166	56.3	1.577	33.0	H^γ 1.257, 1.327, C^γ 24.8; H^δ 1.573, C^δ 29.0; H^ε 2.876, C^ε 41.9; H^ζ 7.470
**L 2**	8.087	122.7	4.348	54.8	1.396 1.486	42.9	H^γ 1.484, C^γ 27.0; $H^{\delta 1}$ 0.873, $C^{\delta 1}$ 24.9; $H^{\delta 2}$ 0.810, $C^{\delta 2}$ 23.4
**W 3**	7.983	120.8	4.927	56.1	2.921	30.9	$H^{\delta 1}$ 7.057, $C^{\delta 1}$ 127.3; $H^{\varepsilon 1}$ 9.958, $N^{\varepsilon 1}$ 129.0; $H^{\varepsilon 3}$ 7.352, $C^{\varepsilon 3}$ 120.8; $H^{\zeta 2}$ 7.445, $C^{\zeta 2}$ 114.4; $H^{\zeta 3}$ 6.894, $C^{\zeta 3}$ 121.9; $H^{\eta 2}$ 7.182, $C^{\eta 2}$ 124.6
**L 4**	8.512	123.2	4.491	54.3	1.455	43.8	H^γ 1.423, C^γ 26.9; $H^{\delta 1}$ 0.864, $C^{\delta 1}$ 24.9; $H^{\delta 2}$ 0.833, $C^{\delta 2}$ 23.9
**W 5**	8.296	121.9	4.975	57.1	3.035 3.349	30.5	$H^{\delta 1}$ 7.194, $C^{\delta 1}$ 127.1; $H^{\varepsilon 1}$ 10.060, $N^{\varepsilon 1}$ 128.8; $H^{\varepsilon 3}$ 7.592, $C^{\varepsilon 3}$ 121.5; $H^{\zeta 2}$ 7.400, $C^{\zeta 2}$ 114.2; $H^{\zeta 3}$ 7.025, $C^{\zeta 3}$ 122.1; $H^{\eta 2}$ 7.137, $C^{\eta 2}$ 124.4
**S 6**	8.832	118.3	4.998	54.6	3.661 3.866	63.4	
**p 7**			4.813	61.5	1.916 2.329	30.5	H^γ 2.041 2.131, C^γ 27.6; H^δ 3.617 3.856, C^δ 50.9
**P 8**			4.545	63.6	2.119 2.267	32.2	H^γ 2.018 2.059, C^γ 26.6; H^δ 3.720, 3.986, C^δ 50.3
**A 9**	7.923	123.9	4.384	51.9	1.409	20.3	
**K 10**	7.961	121.4	4.094	55.7	0.985 1.173	33.0	H^γ 0.603, C^γ 24.4; H^δ 0.920, C^δ 28.9; H^ε 2.119, 2.191, C^ε 41.3; H^ζ 7.086
**L 11**	8.494	125.2	4.465	54.3	1.544	43.5	H^γ 1.528, C^γ 27.0; $H^{\delta 1}$ 0.873, $C^{\delta 1}$ 24.9; $H^{\delta 2}$ 0.835, $C^{\delta 2}$ 23.7
**A 12**	8.288	125.1	4.301	52.2	1.179	19.0	
**V 13**	7.944	119.7	4.048	61.9	1.934	33.0	$H^{\gamma 1}$ 0.844, $C^{\gamma 1}$ 21.1; $H^{\gamma 2}$ 0.789, $C^{\gamma 2}$ 20.6
**E 14**	8.331	124.1	4.305	55.3	1.929 2.073	28.9	H^γ 2.398, 2.435, C^γ 32.7
**amide 15**	7.079 7.552	108.4					

Table S7. Chemical shifts of peptide **7** in water.

Residue	H^N	N	H^α	C^α	H^β	C^β	Others
**Ac 0**			2.015	24.4			
**K 1**	8.211	126.4	4.238	56.2	1.599	33.1	H^γ 1.324, C^γ 24.7; H^δ 1.573, C^δ 28.9; H^ε 2.884, C^ε 41.9; H^ζ 7.484
**S 2**	8.122	116.1	4.408	58.1	3.739	63.8	
**W 3**	7.935	121.6	5.064	55.9	2.939	31.6	$H^{\delta1}$ 7.070, $C^{\delta1}$ 127.5; $H^{\varepsilon1}$ 10.000, $N^{\varepsilon1}$ 129.2; $H^{\varepsilon3}$ 7.185, $C^{\varepsilon3}$ 120.6; $H^{\zeta2}$ 7.435, $C^{\zeta2}$ 114.4; $H^{\zeta3}$ 6.571; $C^{\zeta4}$ 121.7; $H^{\eta2}$ 7.143, $C^{\eta2}$ 124.5
**L^n 4**	8.822	121.6	4.479	55.3	1.659	35.3	H^γ 1.053, 1.127, C^γ 29.3; H^δ 1.248, C^δ 24.7; H^ε 0.840, C^ε 16.2
**W 5**	8.325	120.6	5.149	56.9	3.034<br>3.400	30.9	$H^{\delta1}$ 7.233, $C^{\delta1}$ 127.0; $H^{\varepsilon1}$ 10.000, $N^{\varepsilon1}$ 128.5; $H^{\varepsilon3}$ 7.620, $C^{\varepsilon3}$ 121.7; $H^{\zeta2}$ 7.423, $C^{\zeta2}$ 114.1; $H^{\zeta3}$ 6.996, $C^{\zeta3}$ 122.1; $H^{\eta2}$ 7.130, $C^{\eta2}$ 124.4
**K 6**	9.124	124.9	4.902	53.2	1.614<br>1.889	33.4	H^γ 1.340, 1.430, C^γ 24.9; H^δ 1.706, C^δ 29.3; H^ε 2.969, C^ε 42.0; H^ζ 7.576
**p 7**			4.800	61.4	1.913<br>2.311	30.4	H^γ 2.034, 2.154, C^γ 27.6; H^δ 3.634, 3.865, C^δ 51.0
**P 8**			4.569	63.5	2.163<br>2.249	32.2	H^γ 2.004, 2.075, C^γ 26.4; H^δ 3.731, 3.986, C^δ 50.2
**A 9**	7.888	124.2	4.501	51.6	1.418	20.6	
**K 10**	8.072	122.3	4.223	55.5	0.907<br>1.103	32.9	H^γ 0.385, 0.450, C^γ 24.5; H^δ 0.668, 0.813, C^δ 28.8; H^ε 1.926, 2.028, C^ε 41.1; H^ζ 6.962
**I 11**	8.809	123.8	4.430	59.7	1.835	40.5	$H^{\gamma1}$ 1.065, 1.347, $C^{\gamma1}$ 26.8; $H^{\gamma2}$ 0.882, $C^{\gamma2}$ 17.6; H^δ 0.814, C^δ 13.2
**A 12**	8.423	127.4	4.215	52.4	1.240	18.7	
**A 13**	8.002	124.8	4.206	51.7	1.221	19.7	
**amide 14**	6.998<br>7.455	106.2					

Table S8. Chemical shifts of peptide **8** in water.

Residue	H^N	N	$H^α$	$C^α$	$H^β$	$C^β$	Others
**Ac 0**			2.002	24.4			
**K 1**	8.184		4.193	56.2	1.564	33.1	$H^γ$ 1.277, 1.330, $C^γ$ 24.7; $H^δ$ 1.567, $C^δ$ 28.9; $H^ε$ 2.882, $C^ε$ 41.9; $H^ζ$ 7.496
**S 2**	8.092		4.364	58.0	3.697	63.8	
**W 3**	8.043		4.905	56.3	2.922	31.2	$H^{δ1}$ 7.067, $C^{δ1}$ 127.2; $H^{ε1}$ 9.991; $H^{ε3}$ 7.301, $C^{ε3}$ 120.5; $H^{ζ2}$ 7.425, $C^{ζ2}$ 114.5; $H^{ζ3}$ 6.879, $C^{ζ3}$ 121.8; $H^{η2}$ 7.170, $C^{η2}$ 124.5
**L 4**	8.646		4.503	54.2	1.467	43.8	$H^γ$ 1.383, $C^γ$ 26.9; $H^{δ1}$ 0.828, $H^{δ2}$ 0.848, $C^{δ1}$ 24.0, $C^{δ2}$ 25.0
**W 5**	8.282		5.037	56.7	3.035<br>3.330	30.3	$H^{δ1}$ 7.197, $C^{δ1}$ 126.9; $H^{ε1}$ 10.040; $H^{ε3}$ 7.602, $C^{ε3}$ 121.5; $H^{ζ2}$ 7.391, $C^{ζ2}$ 114.1; $H^{ζ3}$ 7.024, $C^{ζ3}$ 122.1; $H^{η2}$ 7.134, $C^{η2}$ 124.4
**L 6**	8.921		4.951	51.9	1.531<br>1.624	42.6	$H^γ$ 1.531, $C^γ$ 27.0; $H^{δ1}$ 0.913, $C^{δ1}$ 24.2; $H^{δ2}$ 0.918, $C^{δ2}$ 25.0
**p 7**			4.758	61.4	1.911<br>2.300	30.4	$H^γ$ 2.027, 2.141, $C^γ$ 27.7; $H^δ$ 3.663, 3.834, $C^δ$ 50.9
**P 8**			4.553	63.6	2.135<br>2.257	32.2	$H^γ$ 2.004, 2.056, $C^γ$ 26.4; $H^δ$ 3.727, 3.983, $C^δ$ 50.3
**A 9**	7.900		4.449	51.7	1.420	20.4	
**R 10**	8.025		4.250	55.3	0.914<br>1.207	30.6	$H^γ$ 0.777, $C^γ$ 26.6; $H^δ$ 2.235, 2.283, $C^δ$ 42.8; $H^ε$ 6.312
**V 11**	8.591		4.329	61.0	2.034	34.4	$H^{γ1}$ 0.836, $C^{γ1}$ 20.3; $H^{γ2}$ 0.894, $C^{γ2}$ 21.2
**A 12**	8.391		4.154	52.4	1.189	18.8	
**A 13**	8.038		4.198	52.1	1.225	19.3	
**E 14**	8.181		4.283	55.4	1.935<br>2.092	28.9	$H^γ$ 2.426, 2.461, $C^γ$ 32.7
**amide 15**	7.088<br>7.519						

Table S9. Chemical shifts of peptide **9** in water.

Residue	H^N	N	H^α	C^α	H^β	C^β	Others
**Ac 0**			2.014	24.4			
**K 1**	8.241	126.2	4.255	56.3	1.631	33.1	H^γ 1.362, C^γ 24.8; H^δ 1.598, C^δ 28.9; H^ε 2.921, C^ε 41.9; H^ζ 7.504
**S 2**	8.167	115.8	4.393	58.3	3.754	63.8	
**W 3**	7.954	121.3	5.035	56.0	2.927	31.5	$H^{\delta1}$ 7.096, $C^{\delta1}$ 127.4; $H^{\varepsilon1}$ 10.020, $N^{\varepsilon1}$ 129.2; $H^{\varepsilon3}$ 7.191, $C^{\varepsilon3}$ 120.6; $H^{\zeta2}$ 7.435, $C^{\zeta2}$ 114.4; $H^{\zeta3}$ 6.658; $C^{\zeta4}$ 121.8; $H^{\eta2}$ 7.149, $C^{\eta2}$ 124.5
**L^n 4**	8.829	121.7	4.488	55.4	1.658	35.3	H^γ 1.051, 1.119, C^γ 29.3; H^δ 1.245, C^δ 24.7; H^ε 0.836, C^ε 16.2
**W 5**	8.327	120.7	5.127	56.9	3.034<br>3.383	30.8	$H^{\delta1}$ 7.225, $C^{\delta1}$ 127.0; $H^{\varepsilon1}$ 10.080, $N^{\varepsilon1}$ 128.5; $H^{\varepsilon3}$ 7.622, $C^{\varepsilon3}$ 121.7; $H^{\zeta2}$ 7.412, $C^{\zeta2}$ 114.1; $H^{\zeta3}$ 7.001, $C^{\zeta3}$ 122.1; $H^{\eta2}$ 7.133, $C^{\eta2}$ 124.4
**K 6**	9.095	124.9	4.893	53.2	1.610<br>1.886	33.4	H^γ 1.334, 1.425, C^γ 24.9; H^δ 1.702, C^δ 29.3; H^ε 2.963, C^ε 41.9; H^ζ 7.578
**p 7**			4.805	61.4	1.908<br>2.306	30.4	H^γ 2.030, 2.148, C^γ 27.6; H^δ 3.636, 3.861, C^δ 51.0
**P 8**			4.569	63.5	2.156<br>2.244	32.2	H^γ 2.001, 2.071, C^γ 26.4; H^δ 3.729, 3,985, C^δ 50.3
**A 9**	7.886	124.1	4.494	51.6	1.417	20.6	
**K 10**	8.071	122.3	4.249	55.5	0.893<br>1.130	32.9	H^γ 0.463, C^γ 24.5; H^δ 0.729, 0.865, C^δ 28.9; H^ε 1.965, 2.069, C^ε 41.1; H^ζ 6.996
**I 11**	8.796	123.2	4.472	59.8	1.836	40.7	$H^{\gamma1}$ 1.058, 1.334, $C^{\gamma1}$ 26.8; $H^{\gamma2}$ 0.871, $C^{\gamma2}$ 17.7; H^δ 0.803, C^δ 13.2
**R 12**	8.412	124.4	4.133	56.0	1.534<br>1.659	30.3	H^γ 1.324, 1.371, C^γ 26.9; H^δ 2.875, C^δ 42.9; H^ε 7.018, N^ε 121.3
**A 13**	8.058	127.0	4.216	51.7	1.223	19.8	
**amide 14**	6.989<br>7.496	106.5					

Table S10. Chemical shifts of peptide **10** in water.

Residue	H^N	N	H^α	C^α	H^β	C^β	Others
**Ac 1**			1.933	24.3			
**K 2**	8.060	126.4	4.212	56.3	1.628	33.5	H^γ 1.236 1.336, C^γ 24.8; H^δ 1.607, C^δ 29.0; H^ε 2.919, C^ε 41.9; H^ζ 7.527
**W 3**	8.068	120.7	5.060	55.8	2.843 2.899	31.6	$H^{\delta1}$ 7.119, $C^{\delta1}$ 127.5; $H^{\varepsilon1}$ 10.060, $N^{\varepsilon1}$ 129.1; $H^{\varepsilon3}$ 7.179, $C^{\varepsilon3}$ 120.6; $H^{\zeta2}$ 7.440, $C^{\zeta2}$ 114.4; $H^{\zeta3}$ 6.737, $C^{\zeta3}$ 121.9; $H^{\eta2}$ 7.159, $C^{\eta2}$ 124.5
**L^n 4**	9.029	122.3	4.584	55.2	1.708	35.4	H^γ 1.078, 1.165, C^γ 29.3; H^δ 1.269, C^δ 24.7; H^ε 0.847, C^ε 16.2
**W 5**	8.417	120.6	5.174	57.0	3.027 3.387	30.9	$H^{\delta1}$ 7.227, $C^{\delta1}$ 126.9; $H^{\varepsilon1}$ 10.060, $N^{\varepsilon1}$ 128.4; $H^{\varepsilon3}$ 7.628, $C^{\varepsilon3}$ 121.7; $H^{\zeta2}$ 7.394, $C^{\zeta2}$ 114.0; $H^{\zeta3}$ 6.998, $C^{\zeta3}$ 122.1; $H^{\eta2}$ 7.130, $C^{\eta2}$ 124.4
**K 6**	9.181	125.1	4.918	53.1	1.607 1.896	33.5	H^γ 1.341, 1.434, C^γ 24.9; H^δ 1.709, C^δ 29.3; H^ε 2.967, C^ε 41.9; H^ζ 7.586
**p 7**	-	-	4.823	61.4	1.918 2.318	30.4	H^γ 2.040, 2.168, C^γ 27.7; H^δ 3.641, 3.887, C^δ 51.0
**P 8**	-	-	4.598	63.6	2.177 2.256	32.3	H^γ 2.007, 2.084, C^γ 26.4; H^δ 3.740, 4.001, C^δ 50.2
**A 9**	7.871	124.3	4.525	51.6	1.427	20.7	
**K 10**	8.076	122.7	4.256	55.3	0.773 1.078	32.9	H^γ 0.358, 0.417, C^γ 24.4; H^δ 0.717 0.786, C^δ 28.8; H^ε 1.902. 1.966, C^ε 41.0; H^ζ 6.955
**I 11**	8.907	123.5	4.535	59.6	1.829	41.1	$H^{\gamma1}$ 1.038, 1.323, $C^{\gamma1}$ 26.7; $H^{\gamma2}$ 0.870, $C^{\gamma2}$ 17.7; H^δ 0.806, C^δ 13.4
**R 12**	8.440	124.6	4.053	56.0	1.418 1.607	30.2	H^γ 1.210, 1.253, C^γ 27.0; H^δ 2.791, 2.826, C^δ 42.8; H^ε 6.995, N^ε 121.4
**A 13**	8.150	127.6	4.215	51.6	1.218	19.7	
**amide 14**	6.984 7.491	106.7					

Table S11. Chemical shifts of peptide **11** in water.

Residue	H^N	N	H^α	C^α	H^β	C^β	Others
**Ac 1**			1.930	24.3			
**K 2**	8.044	126.4	4.220	56.2	1.584 1.622	33.5	H^γ 1.237 1.332, C^γ 24.9; H^δ 1.604, C^δ 29.0; H^ε 2.918, C^ε 41.9; H^ζ 7.526
**W 3**	8.078	120.9	5.043	55.9	2.833 2.897	31.5	$H^{\delta1}$ 7.121, $C^{\delta1}$ 127.5; $H^{\varepsilon1}$ 10.050, $N^{\varepsilon1}$ 129.1; $H^{\varepsilon3}$ 7.201 $C^{\varepsilon3}$ 120.6; $H^{\zeta2}$ 7.440, $C^{\zeta2}$ 114.4; $H^{\zeta3}$ 6.769; $C^{\zeta3}$ 121.9; $H^{\eta2}$ 7.156, $C^{\eta2}$ 124.5
**L^n 4**	9.007	122.5	4.567	55.3	1.687	35.4	H^γ 1.084, 1.150, C^γ 29.3; H^δ 1.261, C^δ 24.7; H^ε 0.846, C^ε 16.2
**W 5**	8.368	120.7	5.165	56.8	3.021 3.376	30.7	$H^{\delta1}$ 7.211, $C^{\delta1}$ 126.9; $H^{\varepsilon1}$ 10.050, $N^{\varepsilon1}$ 128.3; $H^{\varepsilon3}$ 7.628, $C^{\varepsilon3}$ 121.7; $H^{\zeta2}$ 7.383, $C^{\zeta2}$ 114.0; $H^{\zeta3}$ 7.007, $C^{\zeta3}$ 122.1; $H^{\eta2}$ 7.127, $C^{\eta2}$ 124.3
**L 6**	9.100	126.1	4.991	51.8	1.456 1.759	42.8	H^γ 1.550, C^γ 27.3; $H^{\delta1/2}$ 0.939, $H^{\delta1/2}$ 24.7
**p 7**	-	-	4.795	61.4	1.914 2.309	30.4	H^γ 2. 038, 2.163, C^γ 27.7; H^δ 3.682, 3.882, C^δ 51.0
**P 8**	-	-	4.596	63.6	2.173 2.255	32.3	H^γ 2.006, 2.075, C^γ 26.4; H^δ 3.734, 4.003, C^δ 50.2
**A 9**	7.913	124.4	4.513	51.6	1.440	20.6	
**K 10**	8.030	122.3	4.295	55.3	0.767 1.088	33.0	H^γ 0.380, 0.474, C^γ 24.4; H^δ 0.784, C^δ 28.9; H^ε 1.927, 1.977, C^ε 41.0; H^ζ 6.956
**I 11**	8.879	123.3	4.542	59.6	1.829	41.2	$H^{\gamma1}$ 1.047, 1.327, $C^{\gamma1}$ 26.7; $H^{\gamma2}$ 0.865, $C^{\gamma2}$ 17.7; H^δ 0.816, C^δ 13.4
**R 12**	8.409	124.4	4.055	56.0	1.402 1.596	30.1	H^γ 1.202, 1.238, C^γ 27.0; H^δ 2.778, 2.820, C^δ 42.8; H^ε 6.989, N^ε 121.4
**A 13**	8.139	127.6	4.214	51.6	1.214	19.7	
**amide 14**	6.974 7.500	106.7					

Table S12. Chemical shifts of peptide **12** in water.

Residue	H^N	N	H^α	C^α	H^β	C^β	Others
**Ac 0**			2.038	24.4			
**K 1**	8.247		4.260	56.3	1.686	33.2	H^γ 1.373, 1.414, C^γ 24.8; H^δ 1.660, C^δ 29.0; H^ε 2.966, C^ε 41.9; H^ζ 7.535
**S 2**	8.213		4.407	58.1	3.759	63.8	
**Y3**	8.097		4.658	57.5	2.773<br>2.863	39.5	H^δ 6.983, C^δ 133.3; H^ε 6.770, C^ε 118.0
**L 4**	8.180		4.347	54.6	1.411<br>1.453	43.1	H^γ 1.391, C^γ 26.8; $H^{\delta1}$ 0.818, $C^{\delta1}$ 23.8; $H^{\delta2}$ 0.868, $C^{\delta2}$ 24.9
**Y 5**	8.093		4.791	57.2	2.810<br>3.034	39.2	H^δ 7.066, C^δ 133.3; H^ε 6.769, C^ε 118.0
**L 6**	8.432		4.843	52.2	1.535	42.8	H^γ 1.531, C^γ 26.9; $H^{\delta1}$ 0.901, $C^{\delta1}$ 23.6; $H^{\delta2}$ 0.909, $C^{\delta2}$ 25.2
**p 7**	-		4.712	61.7	1.929<br>2.311	30.5	H^γ 2.035, 2.128, C^γ 27.5; H^δ 3.621 3.716, C^δ 50.7
**P 8**	-		4.462	63.7	2.050<br>2.292	32.1	H^γ 2.042, C^γ 26.7; H^δ 3.716, 3.940, C^δ 50.5
**A 9**	7.845		4.296	52.4	1.434	19.6	
**K 10**	8.032		4.194	56.3	1.529<br>1.687	33.2	H^γ 1.152, 1.199, C^γ 24.8; H^δ 1.506, C^δ 29.2; H^ε 2.768, C^ε 41.7; H^ζ 7.454
**V 11**	8.185		4.113	62.0	2.016	33.4	$H^{\gamma1}$ 0.868, $C^{\gamma1}$ 20.6; $H^{\gamma2}$ 0.901, $C^{\gamma2}$ 21.1
**A 12**	8.319		4.240	52.5	1.357	19.0	
**A 13**	8.095		4.244	52.5	1.311	19.2	
**E 14**	8.155		4.306	55.6	1.986<br>2.131	29.1	H^γ 2.449, 2.477, C^γ 33.3
**amide 15**	7.375<br>7.504						

Table S13. Chemical shifts of peptide **13** in water.

Residue	H^N	N	H^α	C^α	H^β	C^β	Others
**Ac 0**			2.030	24.4			
**K 1**	8.243		4.255	56.3	1.670<br>2.473	33.2	H^γ 1.356, 1.420, C^γ 24.9; H^δ 1.646, 1.682, C^δ 29.0; H^ε 2.960, C^ε 41.9; H^ζ 7.525
**S 2**	8.206		4.402	58.1	3.758	63.8	
**Y 3**	8.095		4.670	57.5	2.764<br>2.854	39.6	H^δ 6.980; H^ε 6.766
**L 4**	8.212		4.357	54.5	1.387<br>1.456	43.2	H^γ 1.391, C^γ 26.9; $H^{\delta1}$ 0.813, $C^{\delta1}$ 23.7; $H^{\delta2}$ 0.860, $C^{\delta2}$ 24.9
**Y 5**	8.113		4.810	57.2	2.801<br>3.038	39.3	H^δ 7.066; H^ε 6.773
**L 6**	8.473		4.853	52.2	1.543	42.8	H^γ 1.545, C^γ 27.0; $H^{\delta1}$ 0.900, $C^{\delta1}$ 23.7; $H^{\delta2}$ 0.903, $C^{\delta2}$ 25.2
**p 7**	-		4.715	61.7	1.917<br>2.300	30.6	H^γ 2.028, 2.117, C^γ 27.6; H^δ 3.609, 3.733, C^δ 50.8
**P 8**	-		4.444	63.7	2.060<br>2.291	32.2	H^γ 2.041, C^γ 26.7; H^δ 3.716, 3.944, C^δ 50.5
**A 9**	7.810		4.318	52.3	1.439	19.6	
**R 10**	8.058		4.211	56.1	1.523<br>1.698	30.9	H^γ 1.363, C^γ 27.0; H^δ 2.906, 2.957, C^δ 43.4; H^ε 6.938
**V 11**	8.222		4.127	61.9	2.031	33.5	$H^{\gamma1}$ 0.904, $C^{\gamma1}$ 21.1; $H^{\gamma2}$ 0.864, $C^{\gamma2}$ 20.5
**A 12**	8.314		4.240	52.5	1.350	19.0	
**A 13**	8.096		4.236	52.5	1.304	19.2	
**E 14**	8.161		4.298	55.6	1.981<br>2.126	29.1	H^γ 2.466, C^γ 33.3
**amide 15**	7.116<br>7.507						

Table S14. Occurrence of cation-π interactions during MD simulations of peptides **2**, **5-11** (expressed as the time percentage during which the distance between the Nζ/Nε of Lys/Arg at position C+2 or C+4 and the center of the benzene ring of the Trp indole moiety at position N-2 or N-4 was shorter than 6 Å (see also Figure S9).

Peptide	cation(C+2)–π(N-4)	cation(C+2)–π(N-2)	cation(C+4)–π(N-4)
**2**	84.2 %	43.9 %	-
**5**	45.1 %	57.5 %	-
**6**	51.9 %	20.6 %	-
**7**	50.1 %	40.1 %	-
**8**	97 %	53.5 %	-
**9**	70.7 %	22.2%	10.4 %
**10**	77.2 %	28.5 %	10.6 %
**11**	0.2 %	62.9 %	94.2 %

Table S15. NMR structure determination statistics of the different peptides

	2	5	6	7
**NMR distance and dihedral constraints**				
Distance restraints				
Total NOE	84	200	95	92
Intra-residue	2	104	28	11
Inter-residue	82	96	67	81
Sequential ($\|i-j\| = 1$)	30	51	36	32
Nonsequential ($\|i-j\| > 1$)	52	45	31	49
Hydrogen bonds	3	3	3	3
**Structure statistics**				
Number of different NOE violations	0	1	0	0
Average number of NOE violations	0	0.1	0	0
Average amount of NOE violation (Å)	0	0.05	0	0
Number of different VdW violations	0	2	1	1
Average number of VdW violations	0	0.2	0.1	0.1
Average amount of VdW violation (Å)	0	0.04	0.02	0.02
Average RMSD* (Å)	1.60	1.76	1.92	1.71

* Pairwise RMSD was calculated among ten refined structures.

Table S15. NMR structure determination statistics of the different peptides (continued)

	8	9	10	11
**NMR distance and dihedral constraints**				
Distance restraints				
Total NOE	111	99	98	97
Intra-residue	17	11	13	13
Inter-residue	94	88	85	84
Sequential ($\|i-j\| = 1$)	39	37	32	38
Nonsequential ($\|i-j\| > 1$)	55	51	53	46
Hydrogen bonds	3	3	3	3
**Structure statistics**				
Number of different NOE violations	0	0	0	0
Average number of NOE violations	0	0	0	0
Average amount of NOE violation (Å)	0	0	0	0
Number of different VdW violations	2	0	0	0
Average number of VdW violations	0.2	0	0	0
Average amount of VdW violation (Å)	0.04	0.	0	0
Average RMSD* (Å)	1.34	1.39	0.64	0.41

* Pairwise RMSD was calculated among ten refined structures.

Table S16. Analytical characterization of the synthetic peptides used in this work (B = β-Ala; X = Nle).

Number	Peptide sequence	$M_{theor.}$[a] (Da)	M_{found}[b] (Da)	t_R[e] (min)
**1**	*Ac*-BKSWLWLNGAKVAAEB-*NH2*	1756.04	1757.401[c]	27.6
**2**	*Ac*-BKSWLWLpPAKVAAEB-*NH2*	1779.12	1780.828[c]	30.1
**3**	*Ac*-KSWLWLpPAKVAAE-*NH2*	1636.96	1635.804[d]	30.8
**4**	*Ac*-KSWLWLpPAKVALE-*NH2*	1679.04	1677.871[d]	23.4
**5**	*Ac*-KSWLWVpPLKLASE-*NH2*	1695.04	1693.877[d]	24.4
**6**	*Ac*-KLWLWSpPAKLAVE-*NH2*	1679.04	1680.279[c]	31.2
**7**	*Ac*-KSWXWKpPAKIAA-*NH2*	1536.89	1538.367[c]	26.0
**8**	*Ac*-KSWLWLpPARVAAE-*NH2*	1664.98	1663.908[d]	31.2
**9**	*Ac*-KSWXWKpPAKIRA-*NH2*	1622.00	1623.482[c]	24.6
**10**	*Ac*-KWXWKpPAKIRA-*NH2*	1534.92	1536.192[c]	24.8
**11**	*Ac*-KWXWLpPAKIRA-*NH2*	1519.91	1521.201[c]	30.6
**12**	*Ac*-KSYLYLpPAKVAAE-*NH2*	1590.89	1592.004[c]	28.3
**13**	*Ac*-KSYLYLpPARVAAE-*NH2*	1618.90	1620.011[c]	28.6
**14**	*Ac*-KSWLWLpPAQVAAE-*NH2*	1636.92	1635.747[d]	32.5

a. Averaged mass
b. Measured by MALDI-TOF-MS
c. Positive mode $(M+H)^+$
d. Negative mode $(M–H)^−$
e. HPLC gradient: 3% B for 8 min. 3-60% in 35 min. with A = 0.06% TFA in water and B = 0.05% TFA in acetonitrile

References

[1] M. Beisswenger, C. Cabrele, *Biochim. Biophys. Acta* **2014**, *1844*, 1675–1683.

[2] C. Roschger, S. Neukirchen, B. Elsasser, M. Schubert, N. Maeding, T. Verwanger, B. Krammer, C. Cabrele, *ChemMedChem* **2017**, *12*, 1497–1503.

[3] a) C. Das, G. A. Naganagowda, I. L. Karle, P. Balaram, *Biopolymers* **2001**, *58*, 335–346; b) L. M. Gierasch, C. M. Deber, V. Madison, C. H. Niu, E. R. Blout, *Biochemistry* **1981**, *20*, 4730–4738.

[4] S. D. Kiewitz, C. Cabrele, *Biopolymers (Pept. Sci.)* **2005**, *80*, 762–774.

[5] R. M. Fesinmeyer, F. M. Hudson, K. A. Olsen, G. W. White, A. Euser, N. H. Andersen, *J. Biomol. NMR* **2005**, *33*, 213–231.

[6] D. S. Wishart, C. G. Bigam, A. Holm, R. S. Hodges, B. D. Sykes, *J. Biomol. NMR* **1995**, *5*, 67–81.

[7] J. A. Marsh, V. K. Singh, Z. Jia, J. D. Forman-Kay, *Prot. Sci.* **2006**, *15*, 2795–2804.

[8] a) S. Laiken, M. Printz, L. C. Craig, *J. Biol. Chem.* **1969**, *244*, 4454–4457; b) A. C. Gibbs, T. C. Bjorndahl, R. S. Hodges, D. S. Wishart, *J. Am. Chem. Soc.* **2002**, *124*, 1203–1213.
